# Reimagining acidic CO_2_ electroreduction via anion-mediated proton transfer

**DOI:** 10.1093/nsr/nwaf334

**Published:** 2025-08-13

**Authors:** Xinyu Wang, Zhitan Wu, Zhiguo Li, Kai Xie, Yaqiong Wu, Yunpei Yue, Penghan Zhu, Zishan Han, Jiachen Gao, Guangyi Jiang, Daliang Han, Jun Huang, Quan-Hong Yang, Zhe Weng

**Affiliations:** Nanoyang Group, Tianjin Key Laboratory of Advanced Carbon and Electrochemical Energy Storage, School of Chemical Engineering and Technology, and Collaborative Innovation Center of Chemical Science and Engineering (Tianjin), Tianjin University, Tianjin 300072, China; Nanoyang Group, Tianjin Key Laboratory of Advanced Carbon and Electrochemical Energy Storage, School of Chemical Engineering and Technology, and Collaborative Innovation Center of Chemical Science and Engineering (Tianjin), Tianjin University, Tianjin 300072, China; Joint School of National University of Singapore and Tianjin University, International Campus of Tianjin University, Fuzhou 350207, China; Department of Chemistry, National University of Singapore, Singapore 117543, Singapore; Nanoyang Group, Tianjin Key Laboratory of Advanced Carbon and Electrochemical Energy Storage, School of Chemical Engineering and Technology, and Collaborative Innovation Center of Chemical Science and Engineering (Tianjin), Tianjin University, Tianjin 300072, China; National Industry-Education Platform of Energy Storage, Tianjin University, Tianjin 300072, China; Nanoyang Group, Tianjin Key Laboratory of Advanced Carbon and Electrochemical Energy Storage, School of Chemical Engineering and Technology, and Collaborative Innovation Center of Chemical Science and Engineering (Tianjin), Tianjin University, Tianjin 300072, China; National Industry-Education Platform of Energy Storage, Tianjin University, Tianjin 300072, China; Nanoyang Group, Tianjin Key Laboratory of Advanced Carbon and Electrochemical Energy Storage, School of Chemical Engineering and Technology, and Collaborative Innovation Center of Chemical Science and Engineering (Tianjin), Tianjin University, Tianjin 300072, China; Nanoyang Group, Tianjin Key Laboratory of Advanced Carbon and Electrochemical Energy Storage, School of Chemical Engineering and Technology, and Collaborative Innovation Center of Chemical Science and Engineering (Tianjin), Tianjin University, Tianjin 300072, China; Nanoyang Group, Tianjin Key Laboratory of Advanced Carbon and Electrochemical Energy Storage, School of Chemical Engineering and Technology, and Collaborative Innovation Center of Chemical Science and Engineering (Tianjin), Tianjin University, Tianjin 300072, China; National Industry-Education Platform of Energy Storage, Tianjin University, Tianjin 300072, China; Nanoyang Group, Tianjin Key Laboratory of Advanced Carbon and Electrochemical Energy Storage, School of Chemical Engineering and Technology, and Collaborative Innovation Center of Chemical Science and Engineering (Tianjin), Tianjin University, Tianjin 300072, China; Nanoyang Group, Tianjin Key Laboratory of Advanced Carbon and Electrochemical Energy Storage, School of Chemical Engineering and Technology, and Collaborative Innovation Center of Chemical Science and Engineering (Tianjin), Tianjin University, Tianjin 300072, China; Nanoyang Group, Tianjin Key Laboratory of Advanced Carbon and Electrochemical Energy Storage, School of Chemical Engineering and Technology, and Collaborative Innovation Center of Chemical Science and Engineering (Tianjin), Tianjin University, Tianjin 300072, China; Nanoyang Group, Tianjin Key Laboratory of Advanced Carbon and Electrochemical Energy Storage, School of Chemical Engineering and Technology, and Collaborative Innovation Center of Chemical Science and Engineering (Tianjin), Tianjin University, Tianjin 300072, China; National Industry-Education Platform of Energy Storage, Tianjin University, Tianjin 300072, China; Haihe Laboratory of Sustainable Chemical Transformations, Tianjin 300192, China; Institute of Energy Technologies, IET-3: Theory and Computation of Energy Materials, Forschungszentrum Jülich GmbH, Jülich 52425, Germany; Theory of Electrocatalytic Interfaces, Faculty of Georesources and Materials Engineering, RWTH Aachen University, Aachen 52062, Germany; Nanoyang Group, Tianjin Key Laboratory of Advanced Carbon and Electrochemical Energy Storage, School of Chemical Engineering and Technology, and Collaborative Innovation Center of Chemical Science and Engineering (Tianjin), Tianjin University, Tianjin 300072, China; Joint School of National University of Singapore and Tianjin University, International Campus of Tianjin University, Fuzhou 350207, China; National Industry-Education Platform of Energy Storage, Tianjin University, Tianjin 300072, China; Haihe Laboratory of Sustainable Chemical Transformations, Tianjin 300192, China; Nanoyang Group, Tianjin Key Laboratory of Advanced Carbon and Electrochemical Energy Storage, School of Chemical Engineering and Technology, and Collaborative Innovation Center of Chemical Science and Engineering (Tianjin), Tianjin University, Tianjin 300072, China; National Industry-Education Platform of Energy Storage, Tianjin University, Tianjin 300072, China; Haihe Laboratory of Sustainable Chemical Transformations, Tianjin 300192, China

**Keywords:** electrocatalyst, CO_2_ electroreduction, interface, electrolyte engineering, anion effect

## Abstract

Acidic CO_2_ electroreduction reaction (CO_2_RR) offers a carbon-negative pathway for synthesizing value-added chemicals with high carbon efficiency but is significantly hindered by the competing hydrogen evolution reaction (HER). While concentrated K^+^ cations have been extensively employed to suppress HER and improve CO_2_RR selectivity, they inevitably trigger catastrophic salt precipitation that degrades the durability of the electrolyzer. Here, we pioneer an anion engineering strategy that breaks the cation-concentration paradigm through manipulating proton transfer dynamics. Combining mass spectrometry, spectroscopic techniques and theoretical calculations, we reveal that hydrolyzable anions improve proton transfer via forming protonated species that simultaneously strengthen hydrogen-bond networks and lower the kinetic barrier for *H intermediate formation, thereby promoting HER. By leveraging this fundamental insight, we achieved highly selective CO_2_RR with 87.3% Faradaic efficiency in strong acidic conditions (pH 1) at a low K^+^ concentration (0.2 M) using non-hydrolyzable Cl^−^. Our work provides a paradigm shift from cation-centric to anion-dominated electrolyte design, and establishes anion hydrolysis tendency as a crucial descriptor of electrocatalytic performance in acidic CO_2_RR systems.

## Teaser

Protonated species generated through anion hydrolysis facilitate proton transfer, uncovering that proton transport in acidic electrolytes, rather than mere pH, constitutes the essential factor governing competitive hydrogen evolution reactions.

## INTRODUCTION

The CO_2_ electroreduction reaction (CO_2_RR) presents a dual-path strategy for closing the carbon cycle and synthesizing value-added chemicals under ambient conditions [1,2]. State-of-the-art CO_2_RR systems predominantly employ neutral/alkaline electrolytes enriched with K^+^ to improve CO_2_ activation kinetics [3,4]. However, these systems suffer from inherent limitations: the reaction between OH^−^ and CO_2_ produces carbonate species [5,6], causing irreversible CO_2_ loss that restricts single-pass carbon efficiency below 25% [7–9]. More critically, the energy-intensive alkaline regeneration process significantly erodes the environmental benefits of this technology [6,9].

Acidic CO_2_RR systems offer an intriguing alternative by circumventing carbonate formation. However, the abundant availability of protons simultaneously exacerbates hydrogen evolution reaction (HER) competition, severely compromising CO_2_RR selectivity [7,10,11]. Recent breakthroughs utilizing ultrahigh K^+^ concentrations (≥3 M) have demonstrated HER suppression through cation-enriched electrical double layers (EDLs) that impede proton transport [10–16]. Nevertheless, such extreme conditions induce catastrophic salt precipitation in gas diffusion electrodes (GDEs) and obstruct CO_2_ permeability [17,18], thus compromising operational stability [19,20]. These challenges underscore the urgent need for electrolyte engineering strategies that decouple HER suppression from cation concentration dependence in acidic media.

Rational electrolyte design requires a systematic understanding of cation-anion-solvent coordination chemistry in modulating interfacial HER dynamics [21–23]. While cation effects have been extensively mapped, the mechanistic role of anions remains shrouded in controversy [24]. Mounting evidence demonstrates that anions critically regulate interfacial microenvironments through three primary aspects: (i) modifying reactant mass transfer [25,26]. In aqueous electrolytes, water molecules and dissolved CO_2_ compete for adsorption sites on the catalyst surface. Hydrophobic anions (e.g. [NTF_2_]^−^) with strong CO_2_ affinity have been reported to enhance CO_2_ mass transport, thereby improving the selectivity of CO_2_RR [27]. (ii) Influencing intermediate adsorption. Specifically adsorbed anions (e.g. halides) have been considered to strengthen, through electronic effects, the binding of key intermediates like *CO, facilitating subsequent reduction steps [28,29]. However, their strong adsorption also reduces the number of active sites for CO_2_RR, potentially reducing CO_2_RR rates [30,31]. (iii) Altering surface pH. Non-buffering anions (e.g. SO_4_^2−^, Cl^−^) demonstrate superior HER inhibition on Cu in neutral media compared to buffering counterparts (e.g. HCO_3_^−^, HPO_4_^2−^), a phenomenon previously attributed to their pH-elevating effects through suppressed buffering [32,33]. However, this interpretation conflicts with observations that elevated pH accelerates HER kinetics on Cu in alkaline electrolytes [34–36]. These seemingly contradictory findings highlight fundamental knowledge gaps in anion-mediated interfacial processes, particularly under acidic CO_2_RR conditions where proton management becomes critically important.

Herein, we systematically investigate anion effects on HER/CO_2_RR competition in acidic media through integrating real-time mass spectrometry, spectroscopic techniques, and theoretical calculations. Our results reveal that non-hydrolyzable anions (ClO_4_^−^, SO_4_^2−^) exhibit superior HER suppression compared to hydrolyzable anions (PO_4_^3−^, OAc^−^), with mechanistic studies demonstrating that this behavior stems from restricted proton transfer rather than surface pH increase. Detailed analysis shows anions undergo protonation (forming H_2_PO_4_^−^, HOAc) that strengthens hydrogen-bond (H-bond) networks and lowers *H formation energy barriers. In contrast, non-hydrolyzable anions create a kinetic bottleneck for proton flux by disrupting continuous H-bond pathways. Leveraging this understanding, we achieved a high CO_2_RR Faradaic efficiency (FE) of 87.3% with a partial current density of 170.4 mA cm^−2^ on commercial Cu catalysts in strongly acidic media (pH 1) using Cl^−^-based electrolytes containing only 0.2 M K^+^, comparable to conventional 3 M K^+^ systems, but with dramatically mitigated salt precipitation and improved operational stability (>12 hours). These findings establish this anion hydrolysis tendency as a crucial design parameter for acidic CO_2_RR systems and provide fundamental insights into proton management strategies to address the persistent competition between CO_2_RR and HER.

## RESULTS AND DISCUSSION

### Effects of anions on competitive HER

**Figure 1.**
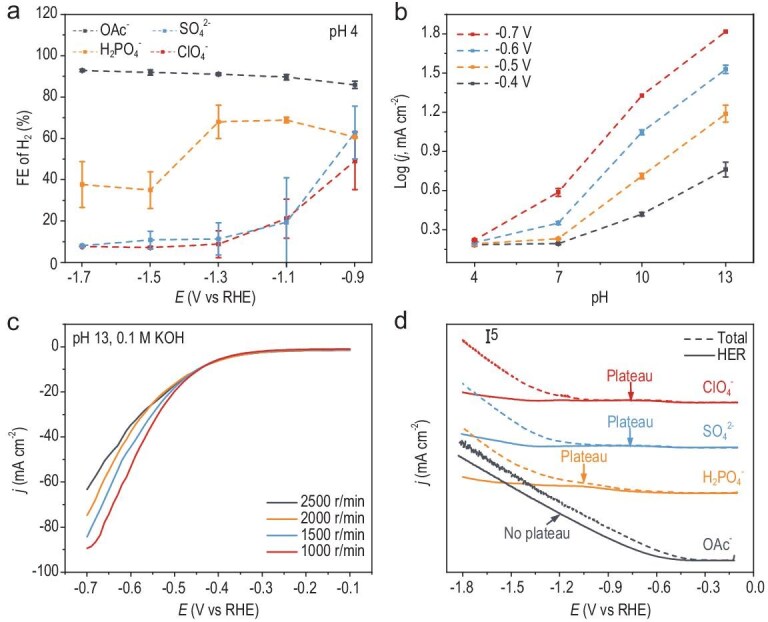
Performance of competitive HER in acidic electrolyte containing different anions. (a) FEs of H_2_ on ED-Cu during chronoamperometry in H-cell under CO_2_ in acidic electrolytes (pH 4): ClO_4_^−^ stands for 0.1 M KClO_4_ (HClO_4_), SO_4_^2−^ stands for 0.05 M K_2_SO_4_ (H_2_SO_4_), H_2_PO_4_^−^ stands for 0.1 M KH_2_PO_4_ (H_3_PO_4_), and OAc^−^ stands for 0.1 M KOAc (HOAc). (b) pH-dependent HER current densities (log scale) at different potentials derived from RDE measurements in Ar-saturated electrolytes containing 0.1 M K^+^ at 50 mV s^−1^ and 2500 rpm. (c) LSV curves of a Cu disk in an Ar-saturated 0.1 M KOH at various rotating speeds. (d) Total current densities and HER current densities in these four electrolytes during LSV on ED-Cu at a scan rate of 1 mV s^−1^. The plateau of HER current is labelled.

We carried out CO_2_ electrolysis using electrodeposited copper (ED-Cu) on a carbon-based substrate (Avcard GDS 2230) in an H-type cell with acidic electrolytes containing 0.1 M K^+^ and different anions (H_2_PO_4_^−^, OAc^−^, ClO_4_^−^ and SO_4_^2−^; see Methods in Supplementary Information for details). Scanning electron microscopy (SEM) images and X-ray diffraction (XRD) patterns confirmed that the ED-Cu is composed of nanostructured Cu particles (Figs S1 and S2). To minimize the interference of bicarbonate/carbonate ions [7,37], the electrolyte pH was initially adjusted to 4, as lowering the pH of a 0.1 M KOAc solution to 3.5 or below by adding HOAc alone proved challenging. The anions were categorized into two groups based on their hydrolysis behavior: hydrolyzable anions (H_2_PO_4_^−^ and OAc^−^) and non-hydrolyzable anions (ClO_4_^−^ and SO_4_^2−^). Hydrolyzable anions tend to exist in the form of protonated species (denoted as HA) under acidic conditions, whereas non-hydrolyzable ones remain fully ionized. For instance, OAc^−^ hydrolyzed to form HOAc and H_2_PO_4_^−^ did not undergo further ionization at pH 4. From −0.9 to −1.7 V (all potentials versus reversible hydrogen electrode (RHE) with 85% iR correction unless specified), ED-Cu exhibited markedly suppressed HER competition and lower partial current densities in the presence of non-hydrolyzable anions compared to hydrolyzable anions (Fig. 1a, Figs S3 and S4). The FE for HER followed a consistent trend across the potential range: ClO_4_^−^ ≈ SO_4_^2−^ < H_2_PO_4_^−^ < OAc^−^, which demonstrates that anions with higher hydrolysis propensity exacerbate HER competition. This phenomenon has also been observed with other metal catalysts, such as gold and silver (Fig. S5).

A reduced hydrolysis tendency leads to elevated surface pH in the diffusion layer compared to bulk electrolyte pH during CO_2_RR, which arises from diminished buffering capacity (Figs S6 and S7), as theoretically verified through protonation equilibrium calculations (Note S1 and Tables S1 and S2) [38]. The increase in pH leads to the depletion of protonated anions and promotes the formation of carbonates on the electrode surface (Fig. S8). To figure out the intrinsic pH-HER correlation without the interference of mass transport effects, we conducted controlled experiments using a Cu rotating disk electrode (RDE) in an Ar-saturated ClO_4_^−^ electrolyte. A high rotation rate of 1000–2500 r/min was used to minimize the diffusion layer thickness to ∼2 μm, ensuring the surface pH closely aligned with that of the bulk electrolyte [39]. Contrary to the conventional wisdom of suppressed HER under more alkaline conditions, linear sweep voltammetry (LSV) shows higher HER current densities with the electrolyte pH increasing from 4 to 13 (Fig. S9). Logarithmic analysis of HER current densities at the highest rotation rate of 2500 r/min revealed a linear pH dependence within the potential window of −0.4 to −0.7 V (Fig. 1b). This finding was further corroborated by the observation that a higher surface pH environment, induced by lowering the rotation speed, leads to a notable enhancement in HER activity (Fig. 1c). While rarely reported in Cu-based CO_2_RR systems [36], the anomalous alkaline-driven HER enhancement may share mechanistic parallels with platinum-based nanomaterials, where surface OH^−^ adsorption facilitates H_2_O dissociation and enhances proton transfer kinetics [40,41]. In contrast, bulk polycrystalline platinum exhibits slower HER kinetics as pH increases. This suggests that the suppression of HER in the presence of non-hydrolyzable anions is primarily driven by proton transfer dynamics rather than the increase in surface pH.

To elucidate the anion-mediated proton transfer dynamics governing HER/CO_2_RR competition, we implemented an integrated analytical approach combining differential electrochemical mass spectrometry (DEMS) with LSV measurements in different electrolytes under CO_2_ atmosphere. DEMS enabled real-time monitoring and quantification of HER activity during LSV scanning from −0.1 V to −1.8 V (Figs S10 and S11) [42]. The HER partial current density served as a kinetic indicator of proton accessibility due to its faster response compared to CO_2_RR, with the appearance of a plateau region in current-potential profiles signifying a limitation in proton supply (Fig. 1d and Fig. S12) [12,43,44]. In the presence of H_2_PO_4_^−^, a transient plateau emerged at −1.1 V, indicating a higher proton-supplying capacity compared to non-hydrolyzable anions (ClO_4_^−^ and SO_4_^2−^), which exhibited an earlier plateau at −0.7 V. Notably, the electrolyte containing OAc^−^ displayed no plateau across the tested potential range, highlighting its superior proton-supplying capability. These results collectively demonstrate that anions with a greater hydrolysis trend significantly enhance proton availability, thereby intensifying HER competitiveness in acidic media, as further analyzed in subsequent mechanistic investigations.

### Hydrolyzable anions reinforce H-bond networks

**Figure 2.**
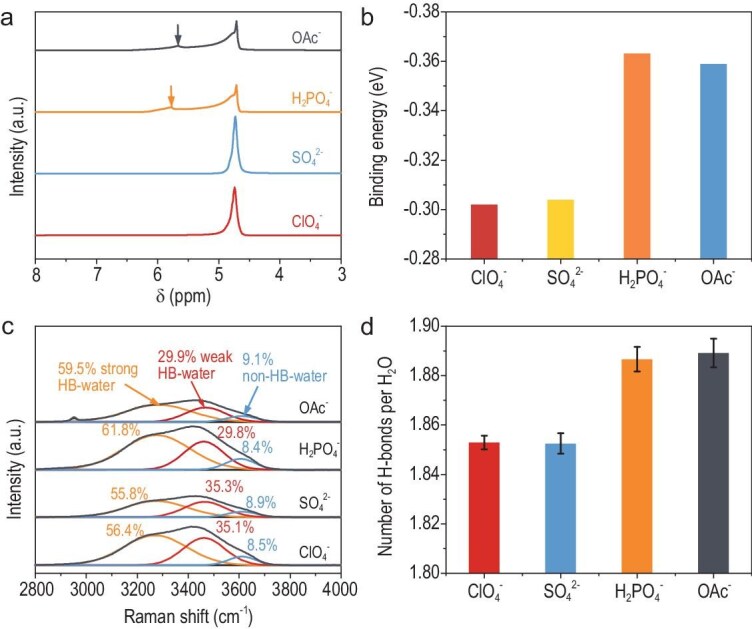
The influence of anions on water structure. (a) ^1^H NMR spectra of acidic electrolytes (pH 4) containing 0.1 M K^+^ with various anions. (b) Relative binding energies between two H_2_O molecules in the presence of ClO_4_^−^, SO_4_^2−^, H_2_PO_4_^2−^, and OAc^−^ (mainly in the hydrolyzed form of HOAc molecules). (c) Raman spectra of the four electrolytes, with the O–H stretching modes fitted to determine the fraction of H_2_O molecules with different H-bonding coordination numbers. (d) Number of H-bonds per H_2_O molecule in the electrolyte box, calculated every 5 ps over a 200 ps molecular dynamics simulation.

Proton transfer through aqueous electrolytes follows the Grotthuß mechanism via concerted H-bond network reorganization (Fig. S13) [45,46]. To probe anion-mediated H-bond modulation, we conducted ^1^H nuclear magnetic resonance (NMR) spectroscopy to examine the chemical environment of H in various electrolytes (Fig. 2a). All spectra exhibited a dominant peak at 4.7 ppm corresponding to bulk H_2_O. The absence of distinct H_3_O^+^ signals arises from rapid proton exchange between H_3_O^+^ and H_2_O, with signal averaging weighted by their relative concentrations. Notably, hydrolyzable anions induced peak broadening, indicative of slowed proton exchange dynamics between HA and H_2_O. Characteristic resonances at 5.8 ppm (H_2_PO_4_^−^) and 5.7 ppm (HOAc) exhibited higher chemical shift values compared to 4.7 ppm for H_2_O, indicating that the H atoms in these species participate in stronger H-bond interactions than those in H_2_O–H_2_O bonds [22]. Density functional theory (DFT) calculations quantified H_2_O–H_2_O interaction energies, showing progressive enhancement from −0.214 eV (PO_4_^3−^) to −0.411 eV (H_3_PO_4_) through sequential protonation (Fig. S14). Comparative analysis of experimental electrolytes demonstrated systematically stronger H_2_O–H_2_O interactions in hydrolyzable anion systems versus non-hydrolyzable analogues (Fig. 2b, Fig. S15 and Table S3).

Raman spectroscopy further corroborated these findings through O–H stretching band deconvolution (Fig. 2c) [47,48]. The presence of hydrolyzable anions increased the fraction of strongly hydrogen-bonded (HB) water on the low-frequency side by ∼5% (absolute), while concurrently reducing the fractions of weakly HB and non-HB species on the high-frequency side. This restructuring elevated total H-bond density by 2.5%, as quantified by molecular dynamics (MD) simulations in a 5.5 nm^3^ electrolyte box (Fig. 2d, Figs S16 and S17). Notably, while HOAc forms H-bonds with H_2_O, their contribution (<1% vs H_2_O–H_2_O bonds) proved negligible (Fig. S18).

These multifaceted analyses establish that HA optimize proton transport via H-bond network reinforcement, rationalizing their HER-enhancing effects in CO_2_RR. However, the comparable binding energy and H-bond number between H_2_O molecules in OAc^−^ and H_2_PO_4_^−^ systems fail to explain their distinct HER activities (Figs 1d, 2b and d), suggesting that additional mechanistic factors govern interfacial proton transfer kinetics.

### Hydrolyzed anions serve as efficient proton donors

**Figure 3.**
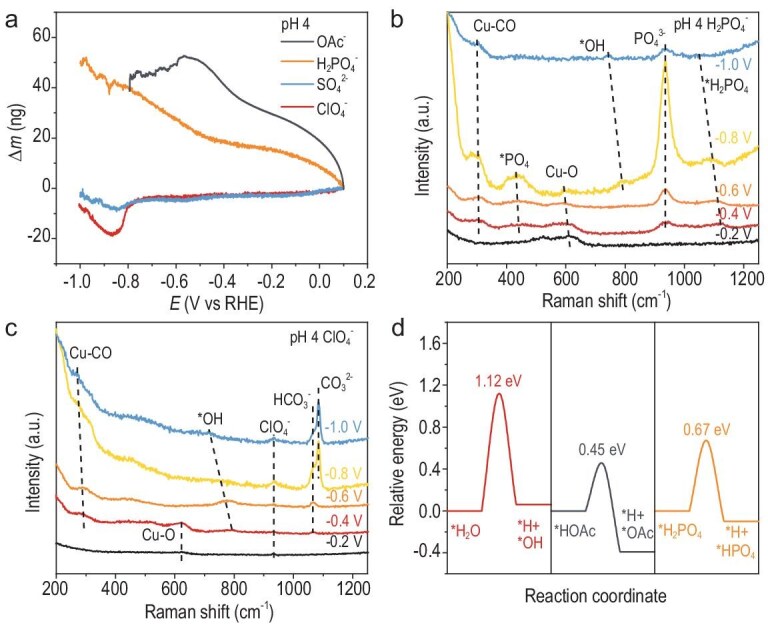
Hydrolyzable anions provide proton donors in acids. (a) Cathode mass changes measured via EQCM during LSV in pH 4: 0.1 M KH_2_PO_4_ (H_3_PO_4_), 0.1 M KClO_4_ (HClO_4_), 0.05 M K_2_SO_4_ (H_2_SO_4_) and 0.1 M KOAc (HOAc). *In-situ* Raman spectra at different applied potentials in pH 4 (b) 0.1 M KH_2_PO_4_ (H_3_PO_4_), (c) 0.1 M KClO_4_ (HClO_4_). (d) Kinetic energy diagram showing the dissociation of different adsorbed protonated species, namely H_2_O, HOAc, H_2_PO_4_^−^, on bare Cu(111) to generate the *H intermediate at −1.0 V vs RHE. The intermediate step corresponds to the transition state.

Proton-donating species at the electrode-electrolyte interface are critical for regulating HER kinetics by mediating *H generation [23,34,49]. During acidic CO_2_RR, H_3_O^+^ depletion shifts proton donor to either HA or H_2_O molecules [43,44]. When the H_3_O^+^ ions are consumed, the hydrolyzable anions enhance the hydrogen-bonding network among water molecules, making them more difficult to dissociate [50,51]. To determine whether negatively charged (e.g. H_2_PO_4_^−^) or neutral (e.g. HOAc) HA species remain adsorbed on the electrode surface under cathodic polarization, we performed electrochemical quartz crystal microbalance (EQCM) measurements to monitor interfacial mass changes during LSV under Ar (Fig. 3a) [27,52]. The observed mass change reflects competing processes: cation adsorption (mass gain) versus anion desorption (mass loss) as potential shifts negatively. In the electrolytes containing non-hydrolyzable anions (ClO_4_^−^ and SO_4_^2−^), predominant anion desorption caused net mass reduction between 0.1 and −0.85 V. Below −0.85 V, a potential exceeding the potential of zero charge (PZC) of low-index Cu facets (∼−0.7 V) [53], complete desorption of physically adsorbed anions permitted cation accumulation, reversing the trend to net mass gain. In contrast, hydrolyzable anions systems (H_2_PO_4_^−^ and HOAc) exhibited significant mass increase across the tested potential range, indicating that the desorption of H_2_PO_4_^−^ and HOAc is markedly hindered.

*In-situ* Raman spectroscopy on ED-Cu identified distinct vibrational bands of phosphate species [54,55]: *H_2_PO_4_ (1123 cm^−1^) and *PO_4_ (441 cm^−1^) displayed Stark effect-induced red shifts with increasing cathodic polarization (Fig. 3b). In contrast, bulk electrolyte species—ClO_4_^−^ (935 cm^−1^), SO_4_^2−^ (1009 cm^−1^) and non-adsorbed PO_4_^3−^ (935 cm^−1^) [55,56]—showed no Stark effect (Fig. 3b, c and Fig. S19). Additionally, the persistent Cu–O vibration at ∼597 cm^−1^ under negative potentials in H_2_PO_4_^−^ electrolytes, which was absent in ClO_4_^−^ electrolytes, suggested the stable formation of oxygen adatoms (Cu–OPO_3_) [54]. These Raman results confirmed the specific adsorption of phosphate species, which inhibits their desorption. For the OAc⁻-containing electrolyte, HOAc molecules neither adsorbed nor desorbed electrostatically but participated in K⁺ solvation (Fig. S20) [57,58]. Collectively, these findings demonstrate that hydrolyzable HA species (H_2_PO_4_^−^ and HOAc) persist near polarized electrodes as interfacial proton donors, while non-hydrolyzable systems rely on H_2_O dissociation for *H generation.

DFT calculations on the Cu(111) surface revealed critical differences in *H generation energetics among various adsorbed proton donors (H_2_O, H_2_PO_4_^−^ and HOAc) (Fig. S21). H_2_O dissociation exhibited an endothermic energy increase of 0.06 eV with a kinetic barrier of 1.12 eV (Fig. 3d), while H_2_PO_4_^−^ demonstrated reduced barriers (Δ*G* = 0.67 eV, Δ*E* = −0.10 eV). Notably, HOAc dissociation became exothermic (Δ*E* = −0.39 eV) with a substantially lower barrier (0.45 eV), rationalizing the superior HER activity in OAc^−^ electrolytes versus H_2_PO_4_^−^ systems. This trend inversely correlated with proton donor acidity (Fig. S22)—lower p*K*_a_ species exhibited enhanced *H formation efficiency.

*In-situ* Raman spectroscopy further corroborated these mechanistic insights: ClO_4_^−^ electrolytes showed HCO_3_^−^ (1066 cm^−1^) and *OH (790 cm^−1^) formation from −0.4 V (Fig. 3c), signaling the dominance of H_2_O dissociation following H_3_O^+^ depletion [5,59]. With further proton consumption, CO_3_^2−^ (1088 cm^−1^) emerged from −0.8 V. Contrastingly, H_2_PO_4_^−^ systems displayed delayed *OH appearance from −0.8 V (Fig. 3b) alongside progressive phosphate adsorption (441 and 1123 cm^−1^ bands intensifying from −0.4 to −0.8 V), confirming H_2_PO_4_^−^ as the primary proton donor prior to H_2_O activation.

These findings establish a self-sustaining proton donor cycle for hydrolyzable anions: surface-bound HA dissociates into *H and deprotonated species (*A), creating concentration gradients that drive HA replenishment from bulk electrolyte (Fig. S23). The negatively charged *A subsequently desorbs and regenerates HA through proton recombination, maintaining continuous *H supply. This mechanism amplifies HER activity through kinetic privileging, underscoring the critical role of anion-speciated proton delivery pathways in governing the selectivity of acidic CO_2_RR.

### CO_2_RR performance in strong acids

**Figure 4.**
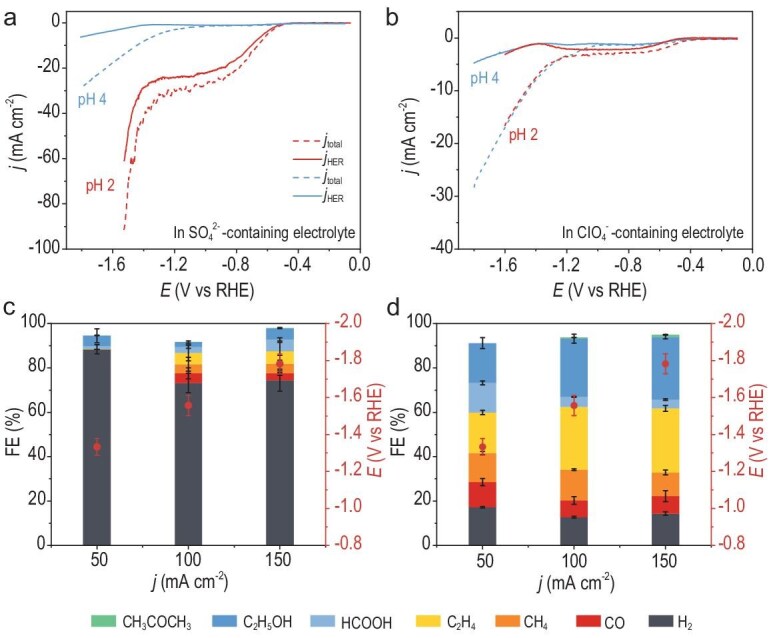
CO_2_RR performance in pH 2 strong acids. Mass spectra of H_2_ in pH 2 (a) 0.05 M K_2_SO_4_ (H_2_SO_4_) and (b) 0.1 M KClO_4_ (HClO_4_) electrolytes during LSV on ED-Cu. FEs of various CO_2_RR products in a flow cell under different applied current densities with pH 2 (c) 0.05 M K_2_SO_4_ (H_2_SO_4_) and (d) 0.1 M KClO_4_ (HClO_4_) electrolytes.

The above mechanistic insights established that efficient CO_2_RR in strong acids necessitates non-hydrolyzable anions to kinetically impede proton transfer. Notably, when the pH decreased from 4 to 2, SO_4_^2−^ underwent significant hydrolysis to HSO_4_^−^ (p*K*_a_ 1.99), which lowers the *H formation energy barrier while strengthening H_2_O–H_2_O interaction (Fig. S24), resulting in a marked increase in HER partial current density during LSV (Fig. 4a) and an FE for HER exceeding 60% (Fig. S25). In contrast, ClO_4_^−^ electrolytes limited HER enhancement under identical conditions (Fig. 4b), consistent with their hydrolysis-resistant nature that limits proton transfer. Flow cell validation demonstrated sustained CO_2_RR selectivity (FE CO_2_RR ≈ 80%) in ClO_4_^−^ electrolytes versus catastrophic HER dominance (FE HER >70%) in HSO_4_^−^ systems (Fig. 4c and d), consistent with the LSV results (Fig. 4a and b). These phenomena further prove that the hydrolysis of anions in acidic media is the main reason for competitive HER.

**Figure 5.**
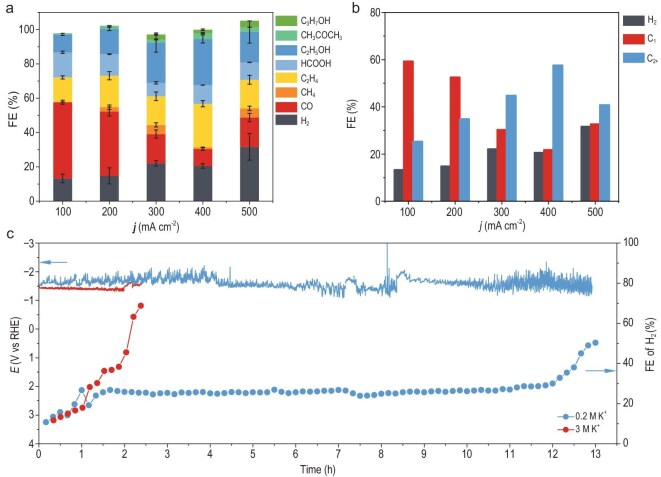
CO_2_RR performance of commercial Cu catalysts in a flow cell with 0.2 M KCl (pH 1). (a) FEs of all products at different applied current densities. (b) Comparison of FEs for C_1_, C_2+_ and H_2_. (c) Stability test at 100 mA cm^−2^ in electrolytes with different K^+^ concentrations, along with the FEs for HER over time.

To validate the universality of our anion-selection strategy in extreme acidic CO_2_RR conditions, we evaluated a commercial Cu catalyst (Figs S26 and S27) under an even stronger acid environment (pH 1, H_3_O^+^ = 0.1 M). Since efficient CO_2_RR require a higher concentration of M^+^ (metal cations) than that of H_3_O^+^ [12], and given the low solubility of KClO_4_ (∼0.1 M at 25°C), we substituted KClO_4_ with KCl (0.2 M, KCl solubility: ∼3.5 M at 25°C) while maintaining a non-hydrolyzable environment. Unlike its CO_2_RR-enhancing role in neutral media [28,29], Cl^−^ exhibited no inherent selectivity modulation in acidic conditions, as evidenced by near-identical CO_2_RR performance in KCl versus KClO_4_ electrolytes (Fig. S28). The 0.2 M KCl electrolyte (pH 1) achieved sustained HER suppression (FE HER <20%) across 100–400 mA cm^−2^ (Fig. 5a). At 200 mA cm^−2^, FE CO_2_RR achieved a peak at 87.3%, rivaling state-of-the-art high-K^+^ acidic systems (Fig. S29). C_2+_ selectivity escalated with current density, reaching 57.6% at 400 mA cm^−2^ (Fig. 5b), with liquid products dominated by HCOOH and ethanol (Fig. S30). While concentrated K^+^ electrolytes (3 M KCl) inhibited HER and improved C_2_H_4_ selectivity (Fig. S31), rapid salt precipitation (Fig. S32) triggered HER dominance within 1 hour (Fig. 5c and Fig. S33) [17,18]. In contrast, the 0.2 M KCl (pH 1) electrolyte demonstrated exceptional stability (>12 hours at 100 mA cm^−2^), which is attributable to its salt precipitation-resistant formulation. After the stability test, the pH of the electrolyte decreased slightly (1.06 to 0.94). This can be attributed to the migration of potassium ions, which, driven by the concentration gradient across the ion-exchange membrane, exchanged with hydrogen ions at the anode.

## CONCLUSIONS

Through a comprehensive investigation of anion effects in acidic CO_2_RR, we demonstrated that non-hydrolyzable anions inhibit HER by introducing additional kinetic barriers for proton transfer, primarily through the disruption of H-bond networks rather than by elevating surface pH. In contrast, hydrolyzable anions enhance proton transfer by forming HA that not only facilitates proton hopping via strengthening the H-bond networks but also mediates *H generation with accelerated dissociation kinetics. Exploiting these principles, we engineered strongly acidic Cl^−^-based electrolytes (pH 1) with low K^+^ concentrations (0.2 M) enabling high CO_2_RR selectivity (87.3% FE) with enhanced operational stability (>12 hours), surpassing conventional 3 M K^+^ systems in both selectivity and salt precipitation resistance. This anion engineering strategy not only resolves the persistent dilemma between carbonate avoidance and HER suppression in acidic CO_2_RR, but also provides a new paradigm for proton management in other proton-coupled electrochemical processes, such as nitrogen reduction reactions and organic electrosynthesis.

## Supplementary Material

nwaf334_Supplemental_File

## Data Availability

Source data are available from the corresponding author upon reasonable request.
