# Supplementary material for: Reimagining acidic CO2 electroreduction via anion-mediated proton transfer
Source: Natl Sci Rev. 2025 Aug 13;12(10):nwaf334. doi: 10.1093/nsr/nwaf334 (PMC12527354; doi:10.1093/nsr/nwaf334)
Supplement: nwaf334_Supplemental_File [file nwaf334_supplemental_file.pdf]

Supplementary Information

Reimagining acidic CO_2 electroreduction via anion-mediated proton transfer

Xinyu Wang[1]‡, *Zhitan Wu*[1,2,5]‡, *Zhiguo Li*[1,3]‡, *Kai Xie*[1,3], *Yaqiong Wu*[1], *Yunpei Yue*[1], *Penghan Zhu*[1,3], *Zishan Han*[1], *Jiachen Gao*[1], *Guangyi Jiang*[1], *Daliang Han*[1,3,4], *Jun Huang*[6,7], *Quan-Hong Yang*[1,2,3,4]* *and Zhe Weng*[1,3,4]*

[1]Nanoyang Group, Tianjin Key Laboratory of Advanced Carbon and Electrochemical Energy Storage, School of Chemical Engineering and Technology, and Collaborative Innovation Center of Chemical Science and Engineering (Tianjin), Tianjin University, Tianjin 300072, China

[2]Joint School of National University of Singapore and Tianjin University, International Campus of Tianjin University, Binhai New City, Fuzhou 350207, China

[3]National Industry-Education Platform of Energy Storage, Tianjin University, Tianjin 300072, China

[4]Haihe Laboratory of Sustainable Chemical Transformations, Tianjin 300192, China

[5]Department of Chemistry, National University of Singapore, 3 Science Drive 3, Singapore 117543

[6]Institute of Energy Technologies, IET-3: Theory and Computation of Energy Materials, Forschungszentrum Jülich GmbH, Jülich 52425, Germany

[7]Theory of Electrocatalytic Interfaces, Faculty of Georesources and Materials Engineering, RWTH Aachen University, Aachen 52062, Germany

*Corresponding Author E-mail: qhyangcn@tju.edu.cn, zweng@tju.edu.cn

‡These authors contributed equally to this work

Methods

Chemicals. K_2SO_4 (>99.0%), KH_2PO_4 (>99.0%), KOAc(>99.0%) and KCl (>99.0%) were purchased from Sinopharm Chemical Reagent Co., Ltd. H_2SO_4 (95–98%), HCl (36–38%), $HClO_4$ (70–72%), H_3PO_4 (85–87%), $KClO_4$ (99.99%), and HOAc (>99.7%) and Cu nanoparticle powder (<100 nm, 99.9% metals basis) were purchased from Aladdin. $CuSO_4$ (99–100.5%) were purchased from Sigma-Aldrich. Aqueous solutions were prepared using deionized (DI) water with a resistivity of 18.2 MΩ cm. All chemicals and materials were purchased from commercial suppliers and used as received, without any further purification.

Preparation of electrolytes. To maintain a consistent K^+ concentration of cathode electrolyte, solution pH was adjusted by mixing two solutions monitored by a pH meter (PHS-3E, Shanghai Leici Instrument Co., Ltd.). For example, Solution A contained 0.1 M $KClO_4$ and 1 M $HClO_4$, while Solution B contained only 0.1 M $KClO_4$. The pH was adjusted to 4 by gradually adding Solution A to Solution B. Electrolytes with different anions and pH values were prepared using similar procedures.

Preparation of working electrodes. ED-Cu was prepared by galvanostatically electrodepositing Cu onto a GDE (Avcard, GDS 2230) for 300 s at 4 mA cm^{-2}. The Cu deposition bath consisted of 0.1 M $CuSO_4$ and 0.005 M H_2SO_4, where the bath solvent was a mixture of EtOH and DI water to improve interface wettability (V_{EtOH}:$V_{DI\ Water}$ = 1:9). For commercial Cu nanoparticles, 10 mg of the Cu nanoparticle powder was added to a mixture of 750 μL of isopropanol, 250 μL DI water and 34 μL of PTFE (10wt% dispersion liquid, Guangdong Canrd New Energy Technology Co.,Ltd.), followed by ultrasonication for 30 min. The obtained ink was then drop-casted onto a GDE, achieving a loading of 1 mg cm^{-2}. After

drying, the electrode was immersed in a 10 wt% PTFE dispersion for 5 seconds. The electrode was then heat-treated in a tube furnace under Ar/H_2 (5% H_2) conditions. The thermal treatment was set to heat from 25 °C to 325 °C at a rate of 5 °C min^{-1}, followed by holding at this temperature for one hour.

Characterizations. Field-emission scanning electron microscopy (FESEM, Hitachi Regulus 8100, 3 kV) was used to characterize the morphology and nanostructure of the samples. The crystal structure was determined using an X-ray diffractometer (XRD, Bruker D8 Focus) with Cu Kα radiation (λ = 1.54056 Å) at 40 kV and 40 mA. XRD spectra were collected over a 2θ range of 30–60° at a scanning speed of 10° min^{-1}. ^{1}H NMR was performed using AVANCE IIITM HD 400 MHz NanoBAY. The water suppression method was used in liquid phase products measurements.

Rotating disk electrode tests. The RDE tests were conducted using a robust rotator (IVI.RRDE, Ivium Tech). Electrochemical measurements were performed in a customized glass cell (Ada Hengsheng Technology Co., Ltd.), where the reference electrode (Ag/AgCl, saturated KCl, Ada Hengsheng Technology) was separated from the disk electrode using a Luggin capillary. The counter electrode (Pt wire) was isolated from the disk electrode with a porous quartz frit. Electrochemical data were recorded using an IviumStat potentiostat (Vertex.C.EIS). The ohmic drop of the electrolyte was determined through electrochemical impedance spectroscopy (EIS) at open circuit potential (OCP), and 85% ohmic drop compensation was applied using IviumSoft software. Before use, the disk electrodes (diameter = 5 mm, Gaossunion Co., Ltd.) were mechanically polished with alumina polishing powders of decreasing sizes: 1.5 μm, 0.5 μm, and 50 nm. The polished disks were then

sonicated in DI water and EtOH (V_{Etoh}:$V_{DI\ Water}$=1:1) for 1 min to remove surface impurities. Argon gas was bubbled through the electrolyte at a flow rate of 20 standard cubic centimeters per minute (sccm).

Electrochemical CO_2RR tests. CO_2RR were performed in a customized two-chamber H-cell (Ada Hengsheng Technology), where CO_2 gas was bubbled directly onto the working electrode. The gas flow rate was maintained at 20 sccm using a mass flow controller (Horiba, SEC-N100). A graphite rod served as the counter electrode, and an Ag/AgCl (saturated KCl) reference electrode (Gaossunion Co., Ltd.) was used for all tests. The cathode and anode chambers were separated by a proton exchange membrane (Nafion^{TM} 117 PEM, DuPont). Electrolysis was conducted using chronoamperometry with a potentiostat (PARSTAT 3000A). An 85% iR compensation was applied by software. For the CO_2RR tests in a three-chamber flow cell (Ada Hengsheng Technology), a GDE and a graphite plate were served as working electrode and counter electrode, respectively. The distance between the cathode and anode was 3 mm. The electrolyte flow rates in both the anode and cathode chambers were adjusted to 6 mL min^{-1}.The working electrode area was 1×1 cm^2. Electroreduction was performed in chronopotentiometry mode. For each specified current density, the products were quantified after 480 seconds of electrolysis. Each experiment was repeated at least three times to obtain average values and standard deviations. All potentials were referenced to RHE with iR correction based on the following formula:

$$E_{RHE} = E_{Ag/AgCl} + 0.197 + 0.059 \times pH + 0.85 \times iR \quad (1)$$

Molecular dynamics simulation. Electrolyte models were constructed, and kinetic calculations were performed using the Forcite module in Materials Studio[1]. The COMPASS

II force field was employed to describe the interactions between ions and molecules. The electrolyte density was set at experimental values, and the simulations were conducted in an isothermal-isobaric (NVT) ensemble at 298.15 K. A time step of 1 fs was applied for 500 ps to fully relax the system, with a Nose-Hoover thermostat maintaining a constant temperature. The system's energy and temperature stabilized after approximately 30 ps. The hydrogen bond analysis was conducted based on two criteria: a donor–acceptor distance (O·H) of ⩽ 2.4 Å, and an O–H·O angle between 90° and 180°.

DFT calculations. All density functional theory (DFT) calculations were performed using the VASP 6.3.2[2]. The exchange-correlation interactions were treated with the Perdew-Burke-Ernzerhof (PBE) functional within the generalized gradient approximation (GGA)[3]. The projector augmented wave (PAW) method was employed to describe the core-valence electron interactions[4], with a plane-wave energy cutoff of 450 eV. The Cu(111) surface was modeled using a 5×5 supercell with three atomic layers, separated by a 20 Å vacuum layer to prevent interactions between periodic images. Structural optimizations were conducted using a 3×3×1 Γ-centered k-point mesh, with energy and force convergence criteria set to 1×10^{-5} eV and 0.01 eV/Å, respectively. The PBE-D3 method was applied to account for van der Waals interactions between water molecules and the substrate[5], with all calculations performed without symmetry constraints. Solvation effects were incorporated through the VASPsol implicit solvation model[6,7], using a dielectric constant (ε) of 78.54 to represent the aqueous environment. The constant-potential method implemented in the CP-VASP code was employed to calculate proton dissociation energy barrier and free energy change[8,9]. Minimum energy pathways and transition state structures were determined using

the climbing image nudged elastic band (CI-NEB) method[10]. The system potential was maintained at −1 V vs. RHE, with a fermi energy convergence criterion of 0.01 eV. During structural optimization, the total electron number was dynamically adjusted to maintain the target electrode potential, ensuring convergence only when the potential reached the predefined value.

The Gibbs free energy change(ΔG) and transition state energy barrier (ΔG‡) were calculated as:

$$\Delta G = G(\mathrm{final}_{*}^{Q2}) - G(\mathrm{initial}_{*}^{Q1}) + (Q2 - Q1)\mu e$$

$$\Delta G^{\ddagger} = G(\mathrm{TS}^{*QTS}) - G(\mathrm{initial}_{*}^{Q1}) + (Q_{\mathrm{TS}} - Q1)\mu e$$

Where $G(\mathrm{initial}_{*}^{Q1})$, $G(\mathrm{TS}^{*QTS})$ and $G(\mathrm{final}_{*}^{Q2})$ represent the free energies of the initial, transition, and final states of the reaction on the Cu(111) electrode at −1 V vs RHE, respectively. Taking the water dissociation reaction ($*H_2O \rightarrow *H+*OH$) as a representative example, the free energy change for proton dissociation is calculated using the following expression:

$$\Delta G = G[*(\mathrm{H+OH})_{*}^{Q2}] - \mathrm{G}[*(\mathrm{H_2O})_{*}^{Q1}] + |e| \cdot U_{\mathrm{SHE}} + (Q2 - Q1)\mu_{\mathrm{e}}$$

where $Q1$ and $Q2$ represent the net charges on the catalyst's electrode surface before and after the reaction, respectively. These values are determined by the following constraint:

$$E_{\mathrm{F}}({}_{*}^{Q1}) = E_{\mathrm{F}}({}_{*}^{Q2}) = \mu_{\mathrm{e}}$$

μ_e is the electron energy, calculated as:

$$\mu_{\mathrm{e}} = \mu_{\mathrm{SHE}} - |e| \cdot U_{\mathrm{SHE}}$$

Where μ_{SHE} is benchmarked to be −4.6 eV for VASPsol. The relationship between U_{SHE} and U_{RHE} is as follows:

$$U_{RHE}=U_{SHE}+0.059\cdot pH$$

U_{SHE} was calculated based on the pH values of different solvents, and μ_e was subsequently derived.

Raman spectroscopy measurements. Raman spectroscopy was performed using a Raman microscopy system (LabRAM HR Evolution, Horiba Jobin Yvon). For electrolyte measurements, a He-Ne laser (λ = 532 nm) was used as the excitation source. For *in-situ* Raman spectroscopy measurements, a laser with λ = 785 nm served as the excitation source. The working electrode was encapsulated in a customized flow cell with a glass window (Gaossunion Co., Ltd., Tianjin). A graphite rod and an Ag/AgCl electrode (saturated KCl) were used as the counter and reference electrodes, respectively.

EQCM measurements. EQCM measurements (Princeton Applied Research, America) paired with LSV were performed on a piezoelectric Cu wafer. The mass change was calculated using a previously reported method[11]. To prevent air exposure, Ar was flowed at 10 sccm over the liquid surface.

Real-time DEMS. The electrolyte was purged with a 20 sccm CO_2 flow in the gas-tight H-type cell. The CO_2 first flowed through the compartment of the working electrode. Gas-phase electrochemical products formed at the working electrode were swept away by the CO_2 flow and directly injected into the mass spectrometer (HPR-20 QIC, R&D, Hiden). Calibration of hydrogen was performed using a previously reported method[12].

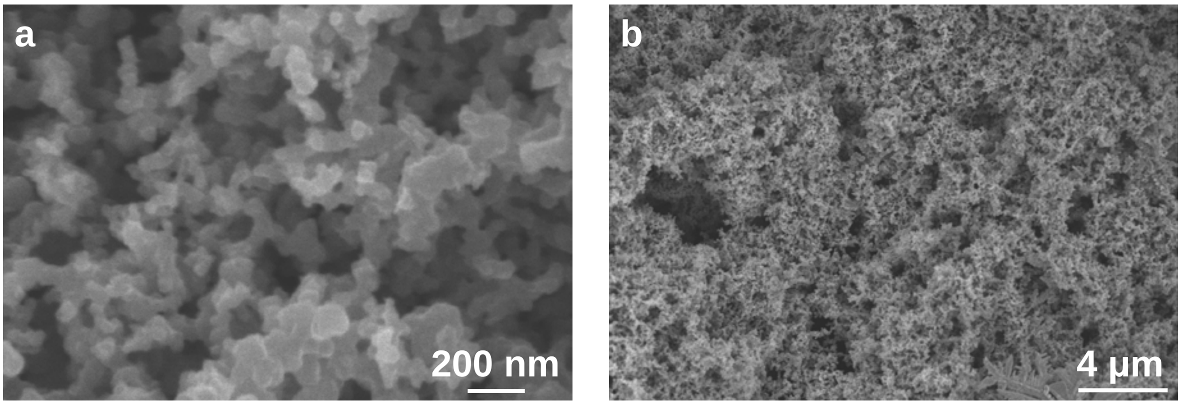

Supplementary Fig. 1 | SEM images of ED-Cu on a commercial GDE.

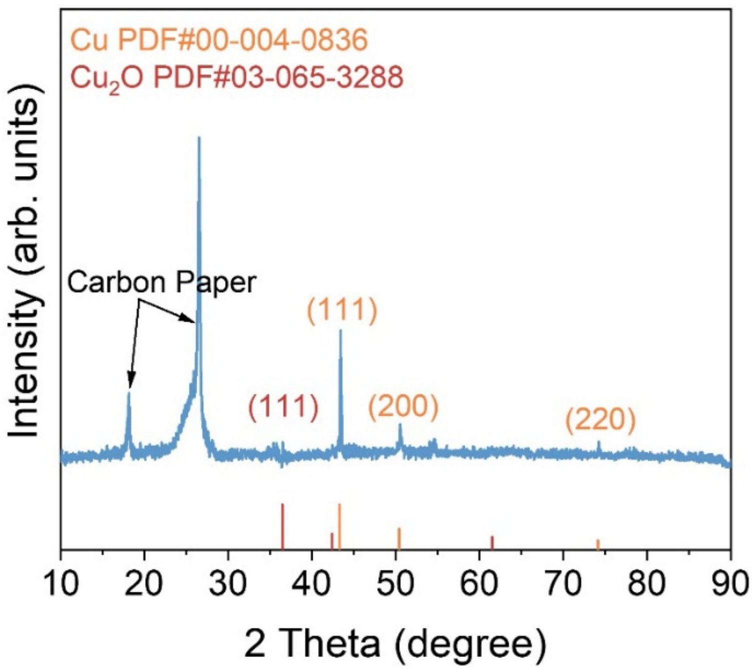

Supplementary Fig. 2 | XRD pattern of ED-Cu on a commercial GDE.

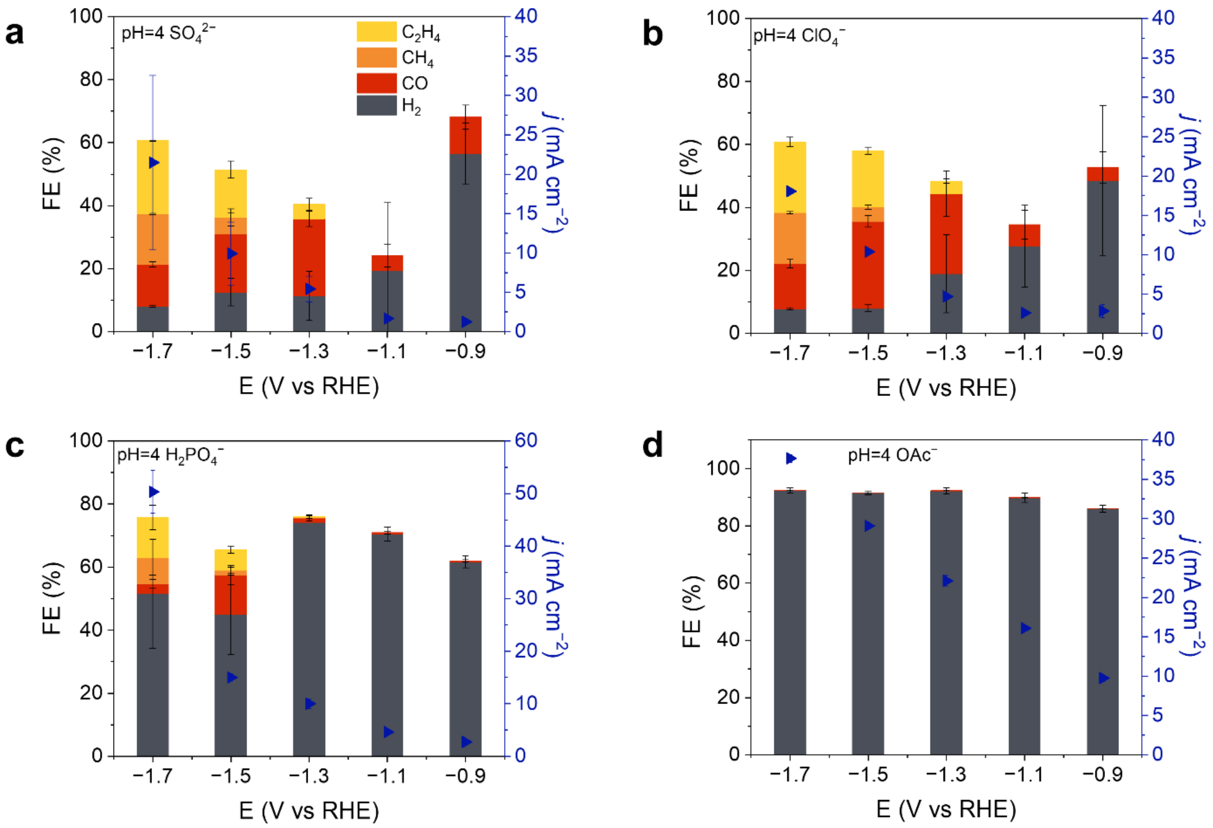

Supplementary Fig. 3 | Total current density and FEs of gas-phase products in acidic electrolytes (pH = 4) with various anions, measured over a potential range of −0.9 to −1.7 V during chronoamperometry in H-cell. (a) pH = 4 0.05 M K_2SO_4 (H_2SO_4), (b) pH = 4 0.1 M $KClO_4$ ($HClO_4$), (c) pH = 4 0.1 M KH_2PO_4 (H_3PO_4), (d) pH = 4 0.1 M KOAc (HOAc).

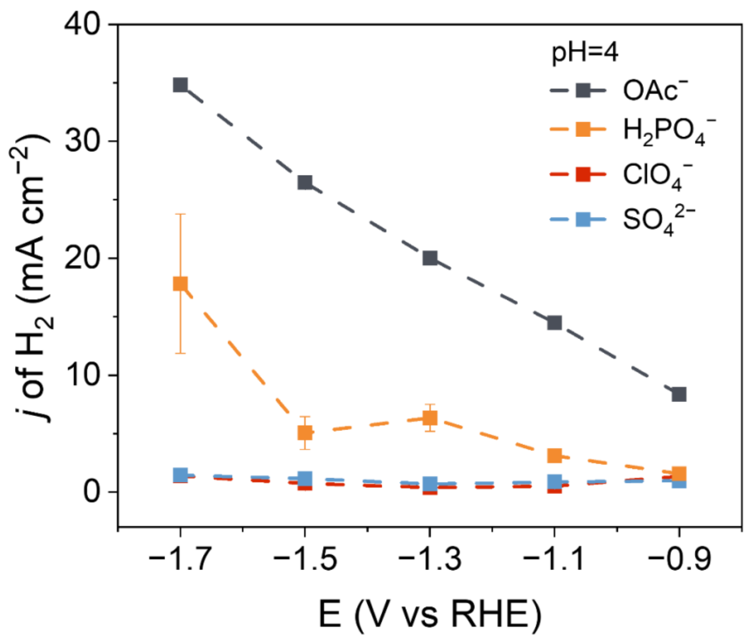

Supplementary Fig. 4 | Partial current densities for HER. Calculated partial current densities of H_2 during CO_2RR in acidic electrolytes (pH = 4) in Supplementary Fig. 3: ClO_4^- stands for 0.1 M $KClO_4$ ($HClO_4$), SO_4^{2-} stands for 0.05 M K_2SO_4 (H_2SO_4), $H_2PO_4^-$ stands for 0.1 M KH_2PO_4 (H_3PO_4), and OAc^- stands for 0.1 M KOAc (HOAc).

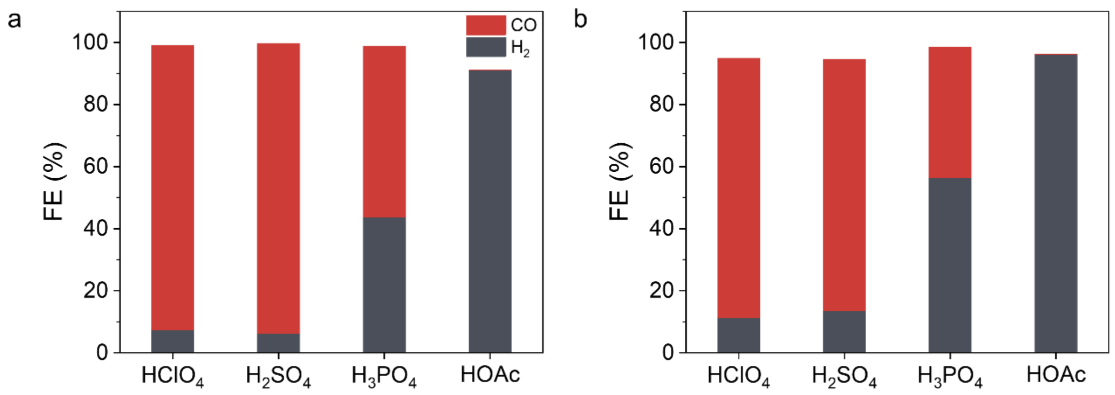

Supplementary Fig. 5 | FEs of gas phase CO_2RR products under −0.9 V vs RHE with pH = 4 acidic electrolytes on (a) Au and (b) Ag.

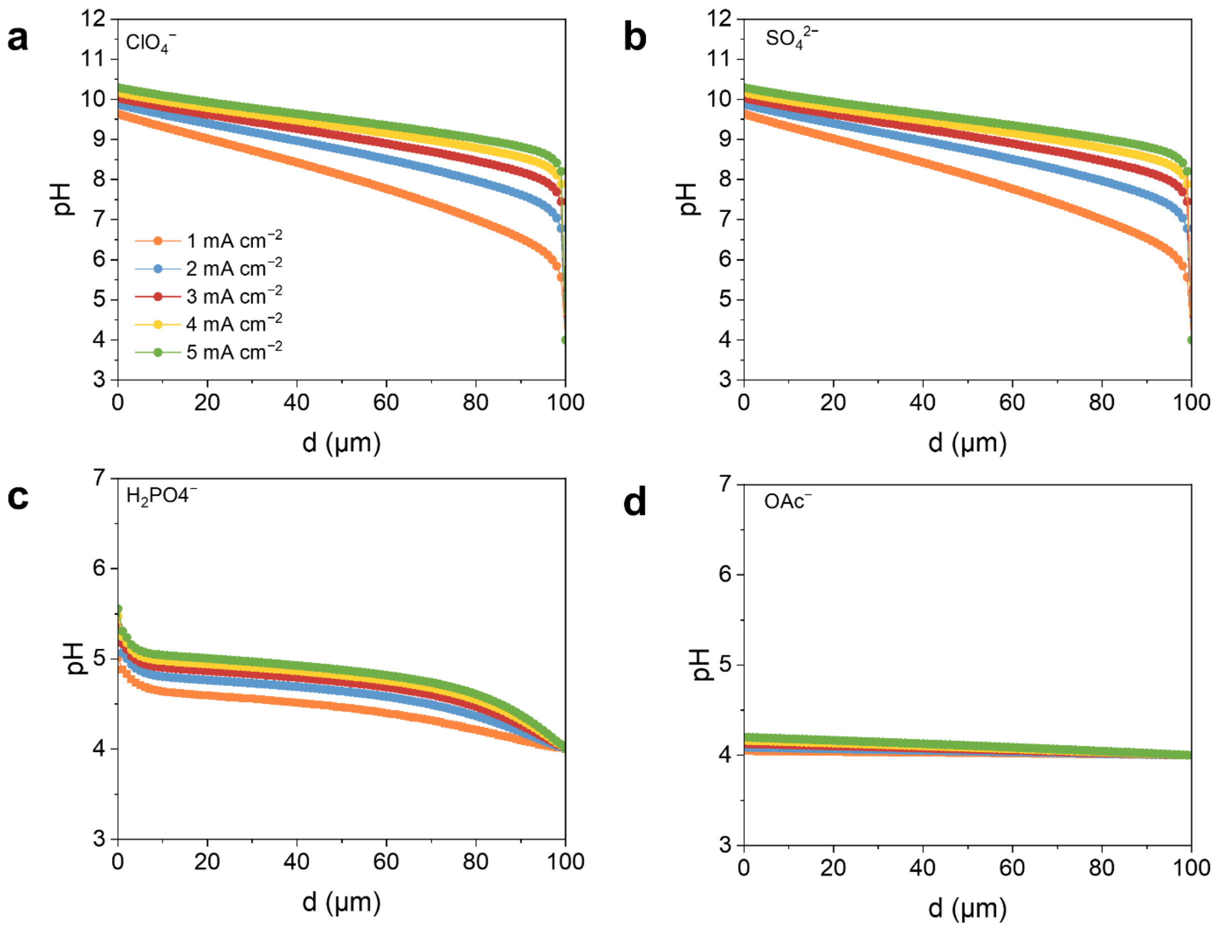

Supplementary Fig. 6 | Calculated pH within a 100 μm diffusion layer under several currents. (a) pH = 4, 0.1 M $KClO_4$ ($HClO_4$), (b) pH = 4, 0.05 M K_2SO_4 (H_2SO_4), (c) pH = 4, 0.1 M KH_2PO_4 (H_3PO_4), and (d) pH = 4, 0.1 M KOAc (HOAc). The x scale represents the distance to the electrode surface.

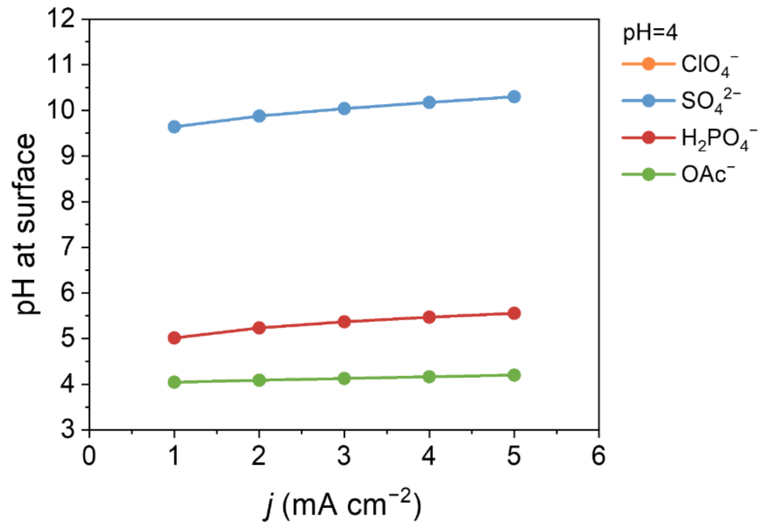

Supplementary Fig. 7 | Calculated surface pH. The pH values were obtained from Supplementary Fig. 5 at x = 0 μm. The line for 0.1 M $KClO_4$ ($HClO_4$) overlaps with the line for 0.05 M K_2SO_4 (H_2SO_4) due to identical numerical modeling results.

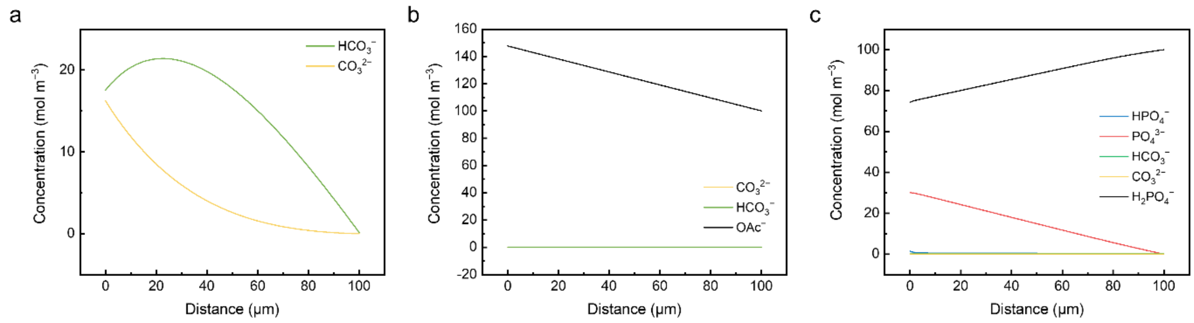

Supplementary Fig. 8 | Surface concentration of anion species under 5 mA cm^{-2} in (a) pH=4 0.1M $KClO_4$ ($HClO_4$), (b) pH=4 0.1M KOAc (HOAc), and (c) pH=4 0.1M KH_2PO_4 (H_3PO_4). The situation for 0.05 M K_2SO_4 (H_2SO_4) is the same as that for 0.1 M $KClO_4$ ($HClO_4$), due to identical numerical modeling results.

Supplementary Note 1

Modeling of local pH at the electrode surface: A modified reaction and diffusion model is used to predict the pH in the diffusion layer, which is adopted from previous works[13-16]. The model takes into account interactions between the following species in the electrolyte containing non-hydrolyzing anions (dissolved CO_2, HCO_3^-, CO_3^{2-}, OH^-), as well as the production of OH^- at the catalyst-electrolyte interface. Dissolved CO_2 is always in equilibrium with the carbonate species in the diffusion layer.

$$CO_2 + OH^- = HCO_3^- \qquad K_1 = 4.44 \times 10^7 \frac{L}{mol} \quad (1)$$

$$HCO_3^- + OH^- = CO_3^{2-} + H_2O \qquad K_2 = 4.66 \times 10^3 \frac{L}{mol} \quad (2)$$

The following equations govern these interactions:

$$\frac{\partial [CO_2]}{\partial t} = D_{CO_2} \frac{\partial^2 [CO_2]}{\partial x^2} - k_{1f}[CO_2][OH^-] + k_{1r}[HCO_3{}^-] + R_{CO_2} \quad (3)$$

$$\frac{\partial [HCO_3^-]}{\partial t} = D_{HCO_3^-} \frac{\partial^2 [HCO_3^-]}{\partial x^2} + k_{1f}[CO_2][OH^-] - k_{1r}[HCO_3{}^-] - k_{2f}[HCO_3{}^-][OH^-] + k_{2r}[CO_3^{2-}] \quad (4)$$

$$\frac{\partial [CO_3^{2-}]}{\partial t} = D_{CO_3^{2-}} \frac{\partial^2 [CO_3^{2-}]}{\partial x^2} + k_{2f}[HCO_3{}^-][OH^-] - k_{2r}[CO_3^{2-}] \quad (5)$$

$$\frac{\partial [OH^-]}{\partial t} = D_{OH^-} \frac{\partial^2 [OH^-]}{\partial x^2} - k_{1f}[CO_2][OH^-] + k_{1r}[HCO_3{}^-] - k_{2f}[HCO_3{}^-][OH^-] + k_{2r}[CO_3^{2-}] + R_{OH} \quad (6)$$

where R_{OH} represents the production of OH^-. k_f and k_r are the rate constants for the forward and reverse reactions and are given in **Supplementary Table 1**. *D* represents the diffusion coefficients at infinite dilution in water for various species, which are obtained from the *Handbook of Chemistry and Physics*[17]. The equilibrium constants for reactions K_1–K_6 were obtained from the literature[13-14]. A catalyst-electrolyte interface is assumed at $x = 0$ μm. A diffusion layer of $x = 100$ μm was assumed in our simulation. The following boundary

conditions were used to solve the coupled equations. The initial values of the concentration were set according to the bulk solution with no current flow. Under current flow, for OH^-, HCO_3^- and CO_3^{2-}, no-flux boundary conditions were applied at the left boundary, while the concentrations at the right boundary were set to the equilibrium values in the bulk solution. The left boundary condition for CO_2 was set according to the bulk solution with no current flow, while a no-flux boundary condition was applied for CO_2 at the right-hand boundary.

For the electrolyte containing non-hydrolyzing anions, the interaction between anions is also considered. For example, in the (pH = 4) 0.1 M KH_2PO_4 (H_3PO_4) electrolyte:

$$H_2PO_4^- + OH^- = HPO_4^{2-} + H_2O \qquad K_4 = 6.17 * 10^6\ L/mol \qquad (7)$$

$$HPO_4^{2-} + OH^- = PO_4^{3-} + H_2O \qquad K_5 = 4.19 * 10\ L/mol \qquad (8)$$

$$\frac{\partial[H_2PO_4^-]}{\partial t} = D_{H_2PO_4^-}\frac{\partial^2[H_2PO_4^-]}{\partial x^2} - k_{4f}[H_2PO_4{}^-][OH^-] + k_{4r}[HPO_4^{2-}] \qquad (9)$$

$$\frac{\partial[HPO_4^{2-}]}{\partial t} = D_{HPO_4^{2-}}\frac{\partial^2[HPO_4^{2-}]}{\partial x^2} + k_{4f}[H_2PO_4{}^-][OH^-] - k_{4r}[HPO_4^-]$$
$$- k_{5f}[HPO_4{}^{2-}][OH^-] + k_{5r}[PO_4^{3-}] \qquad (10)$$

$$\frac{\partial[OH^-]}{\partial t} = D_{OH^-}\frac{\partial^2[OH^-]}{\partial x^2} - k_{1f}[CO_2][OH^-] + k_{1r}[HCO_3{}^-] - k_{2f}[HCO_3{}^-][OH^-]$$
$$+ k_{2r}[CO_3^{2-}] - k_{4f}[H_2PO_4{}^-][OH^-] + k_{4r}[HPO_4^{2-}]$$
$$- k_{5f}[HPO_4{}^{2-}][OH^-] + k_{5r}[PO_4^{3-}] + R_{OH} \qquad (11)$$

For the 0.1M KOAc (HOAc) electrolyte,

$$HOAc + OH^- = OAc^- + H_2O \qquad K_6 = 1.75 * 10^9\ L/mol \qquad (12)$$

$$\frac{\partial[HOAc]}{\partial t} = D_{HOAc}\frac{\partial^2 y}{\partial x^2} - k_{6f}[HOAc][OH^-] + k_{6r}[OAc^-] \qquad (13)$$

$$\frac{\partial[OAc^-]}{\partial t} = D_{OAc^-}\frac{\partial^2 y}{\partial x^2} + k_{6f}[HOAc][OH^-] - k_{6r}[OAc^-] \qquad (14)$$

$$\frac{\partial[OH^-]}{\partial t} = D_{OH^-}\frac{\partial^2 y}{\partial x^2} - k_{1f}[CO_2][OH^-] + k_{1r}[HCO_3{}^-] - k_{2f}[HCO_3{}^-][OH^-]$$
$$+ k_{2r}[CO_3^{2-}] - k_{6f}[HOAc][OH^-] + k_{6r}[OAc^-] \qquad (15)$$

The consumption of protons at the electrode was determined by the currents. Accordingly, the generation of OH^- may be calculated as follows:

$$R_{OH^-} = \frac{j}{F} \quad (16)$$

$$R_{CO_2} = \frac{j}{F}\left(\frac{FE_{CO}}{z_{CO}} + \frac{FE_{CH_4}}{z_{CH_4}} + 2\frac{FE_{C_2H_4}}{z_{C_2H_4}} + 2\frac{FE_{EtOH}}{z_{EtOH}} + \frac{FE_{HCOOH}}{z_{HCOOH}}\right) \quad (17)$$

where j is the planar current density, z is the number of electrons transferred in electrochemical CO_2RR, and F is Faraday's constant (96486 C mol^{-1}).

Supplementary Table 1. Rate constants for reactions at 25 °C[13].

Reaction	Forward rate constant (k_f)/ M^{-1} s^{-1}	Reverse rate constant (k_r)/ s^{-1}
(1)	5.93E3	1.34E−4
(2)	1E8	2.15E4
(7)	5E10 (assumed)	8.13E3
(8)	5E10 (assumed)	1.19E9
(12)	1E13 (assumed)	5.71E3

Supplementary Table 2. Diffusion coefficients used in the simulation.

Species	Diffusion coefficients	Species	Diffusion coefficients
CO_2	1.91E−9 m^2/s	$H_2PO_4^-$	9.59E−10 m^2/s
H^+	9.311E−9 m^2/s	HPO_4^{2-}	7.59E−10 m^2/s
OH^-	5.73E−9 m^2/s	PO_4^{3-}	8.24E−10 m^2/s
HCO_3^-	1.19E−9 m^2/s	H_3PO_4	9.1E−10 m^2/s
CO_3^{2-}	9.23E−10 m^2/s		

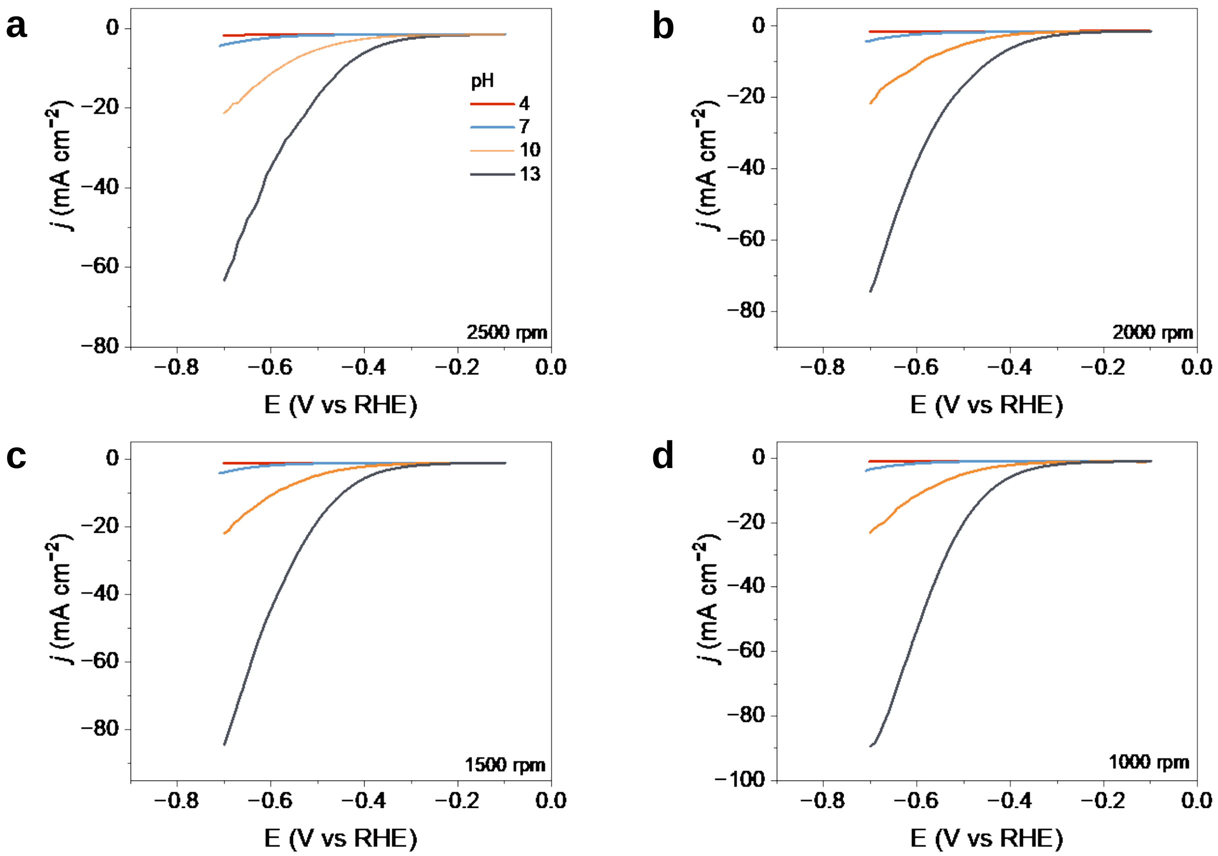

Supplementary Fig. 9 | LSV curves of a Cu disk in the electrolytes containing 0.1 M K^+ at various pH values under Ar, with rotation speeds of (a) 2500 rpm, (b) 2000 rpm, (c) 1500 rpm, and (d) 1000 rpm. The scan rate was 50 mV s^{-1}. At pH = 4, the electrolyte is 0.1 M $KClO_4$+ 0.0001 M $HClO_4$. At pH = 7, the electrolyte is 0.1 M $KClO_4$. At pH = 10, the electrolyte is 0.9999 M $KClO_4$+ 0.0001 M KOH. At pH = 13, the electrolyte is 0.1 M KOH.

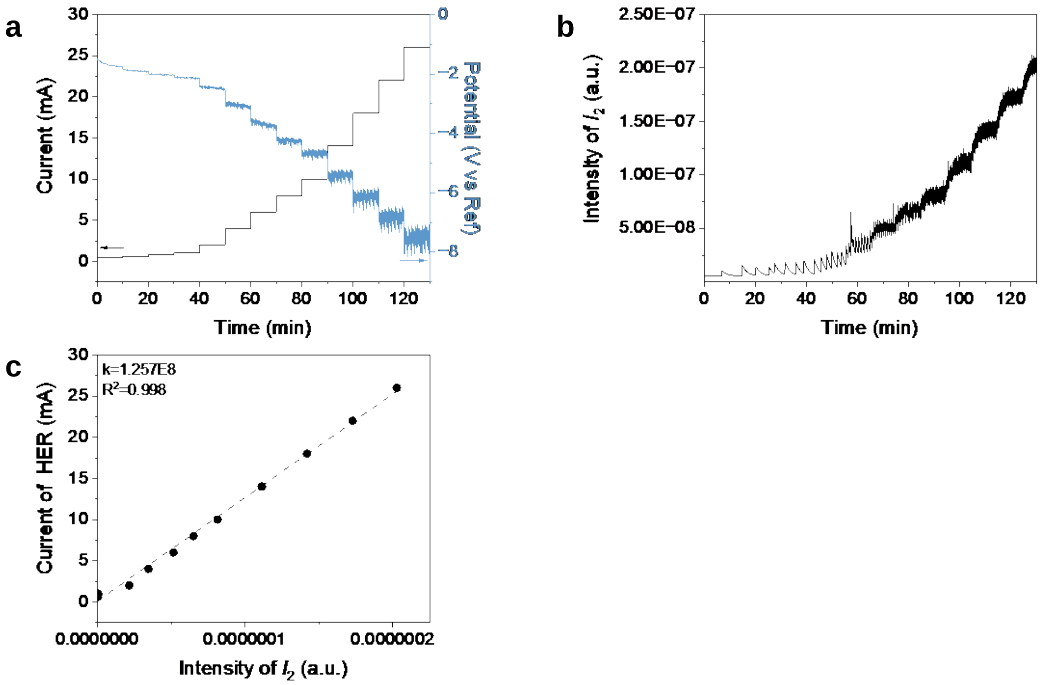

Supplementary Fig. 10 | The calibration curve for hydrogen (H_2) quantification. (a) Multi-step chronopotentiometry curve in a 0.5 M H_2SO_4 solution using a polycrystalline Pt electrode and (b) corresponding mass spectrometry signal in the ionic current for mass 2 (I_2) which indicates H_2. (c) Linearly fitted calibration curve showing the relationship between HER current and I_2 signal intensity.

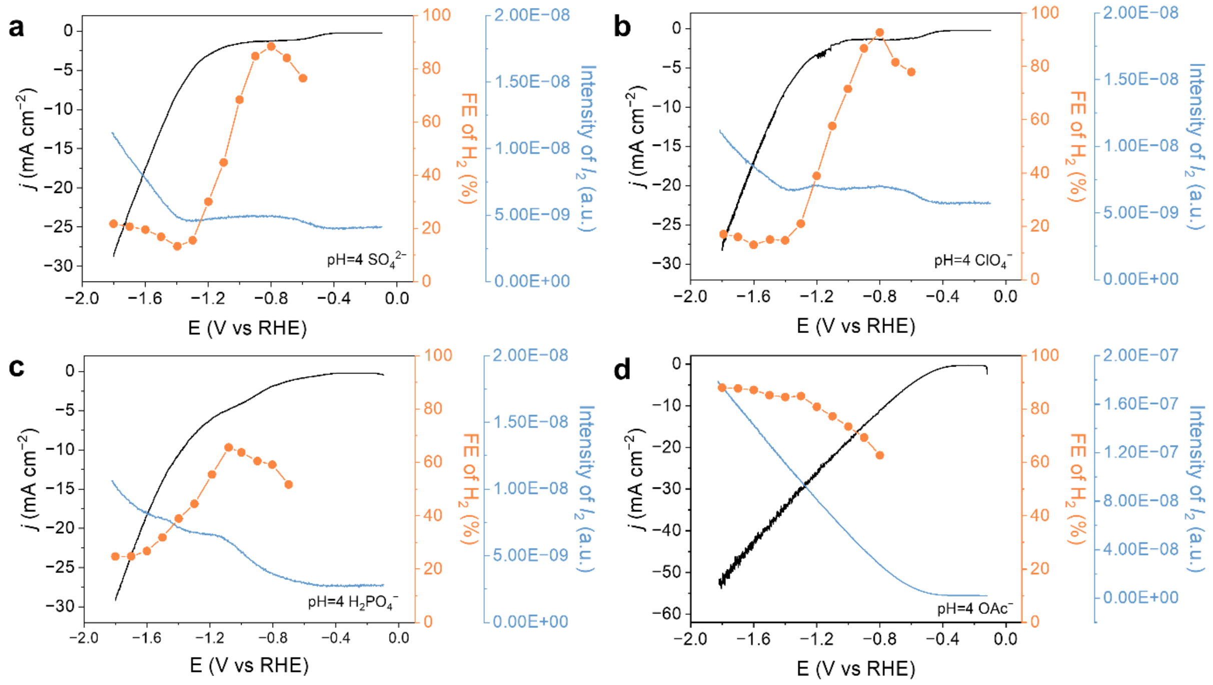

Supplementary Fig. 11 | Total current densities, mass spectra and the corresponding FEs of H_2 in different acidic electrolytes (pH = 4) during LSV. (a) 0.05 M K_2SO_4 (H_2SO_4), (b) 0.1 M $KClO_4$ ($HClO_4$), (c) 0.1 M KH_2PO_4 (H_3PO_4), and (d) 0.1 M KOAc (HOAc). The scan rate of LSV was 1 mV s^{-1}.

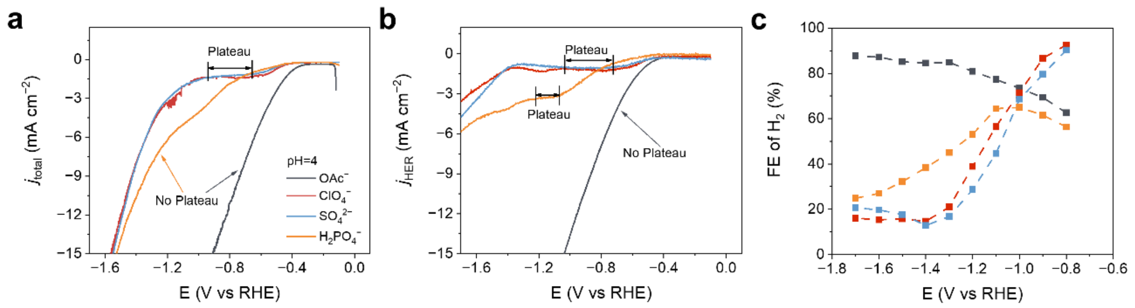

Supplementary Fig. 12 | The influence of anions on proton accessibility. (a) The LSV curves of ED-Cu under CO_2 in acidic electrolytes (pH = 4): 0.1 M $KClO_4$ ($HClO_4$), 0.05 M K_2SO_4 (H_2SO_4), 0.1 M KH_2PO_4 (H_3PO_4), and 0.1 M KOAc (HOAc). (b) The partial current densities and (c) FEs of HER calculated from DEMS.

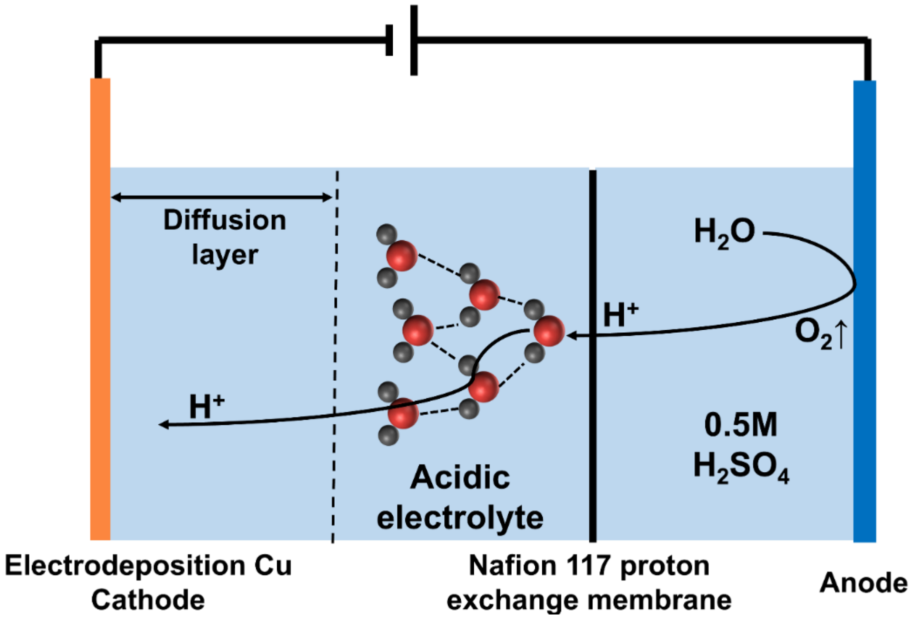

Supplementary Fig. 13 | Schematic diagram of proton transport in acidic CO_2RR electrolyzer, illustrating H^+ generation through the oxygen evolution reaction at the anode.

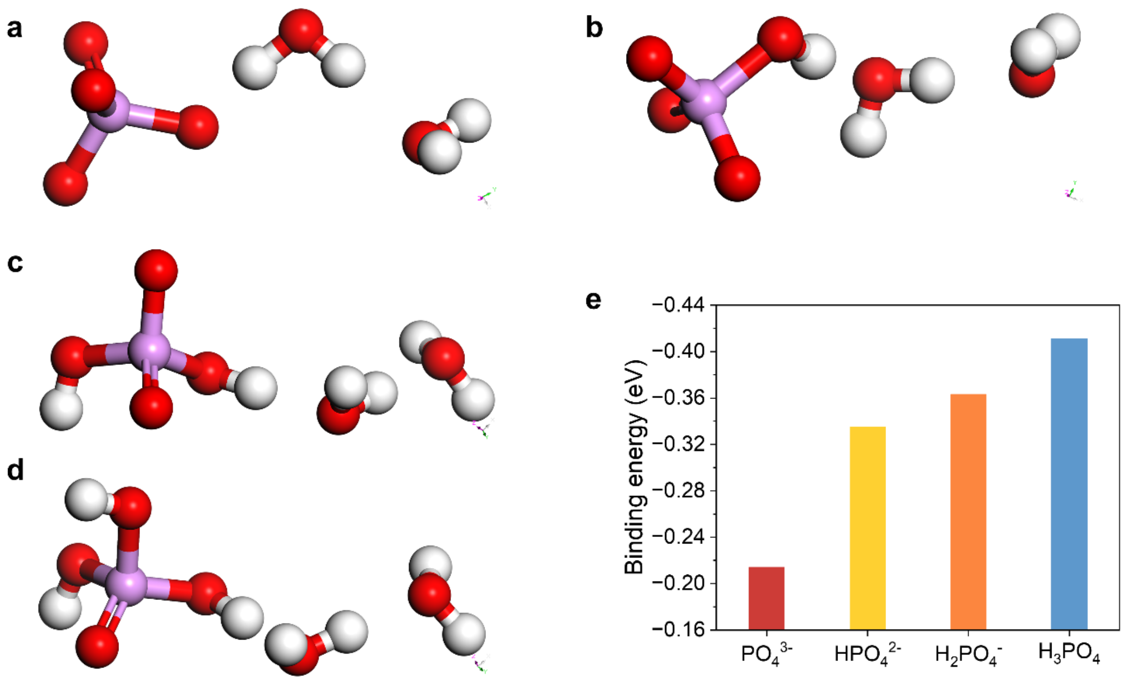

Supplementary Fig. 14 | Anion hydrolysis promotes the interaction between water molecules. (a-c) Atomic structures for calculating interactions between two water molecules in aqueous electrolytes containing (a) a PO_4^{3-} anion, (b) a HPO_4^{2-} anion, (c) a $H_2PO_4^{-}$ anion, and (d) a H_3PO_4 molecule. (e) Relative binding energies between H_2O molecules in the presence of these species. A solvation model was used. The dielectric constant of water was set to 78.54. Red balls stand for oxygen, white balls stand for hydrogen, pink balls stand for phosphorus.

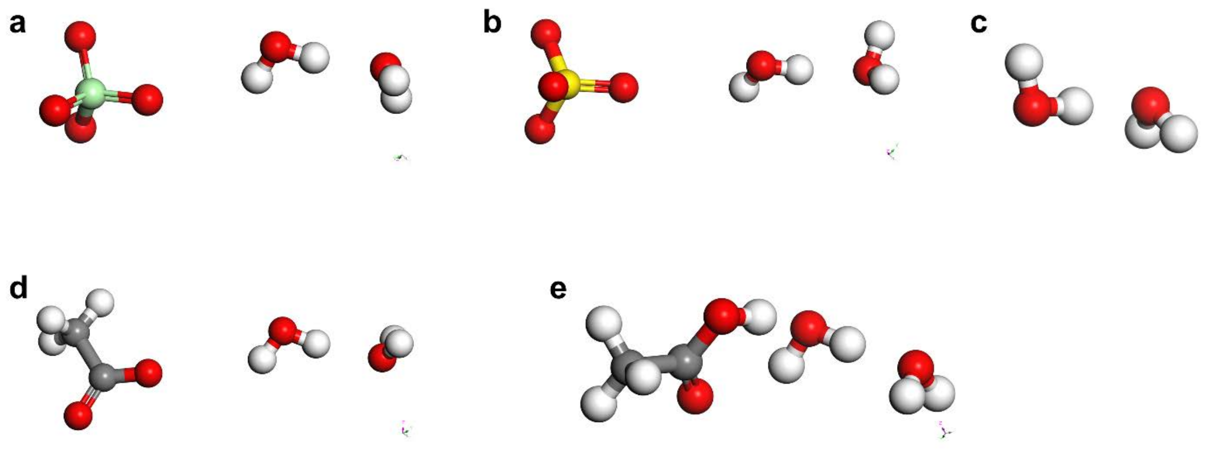

Supplementary Fig. 15 | Atomic structures for calculating interactions between two water molecules. In aqueous electrolytes containing (a) ClO_4^-, (b) SO_4^{2-}, (c) pure water, (d) OAc^-, and (e) HOAc. The dielectric constant of water was set to 78.54. At pH = 4, the hydrolysis of OAc^- is enhanced, leading to the majority of acetate species existing as HOAc molecules. And phosphate species exist mainly in the form of $H_2PO_4^-$. Red balls stand for oxygen, white balls stand for hydrogen, grey balls stand for carbon, green balls stand for chlorine, yellow balls stand for sulfur, pink balls stand for phosphorus.

Supplementary Table 3. Interaction energy of H_2O–H_2O with an anion or a molecule beside.

Species	Interaction energy (E_{int})/ eV
none	−0.246
ClO_4^-	−0.302
SO_4^{2-}	−0.304
PO_4^{3-}	−0.214
HPO_4^-	−0.335
$H_2PO_4^-$	−0.363
H_3PO_4	−0.411
OAc^-	−0.287
HOAc	−0.359
HSO_4^-	−0.370

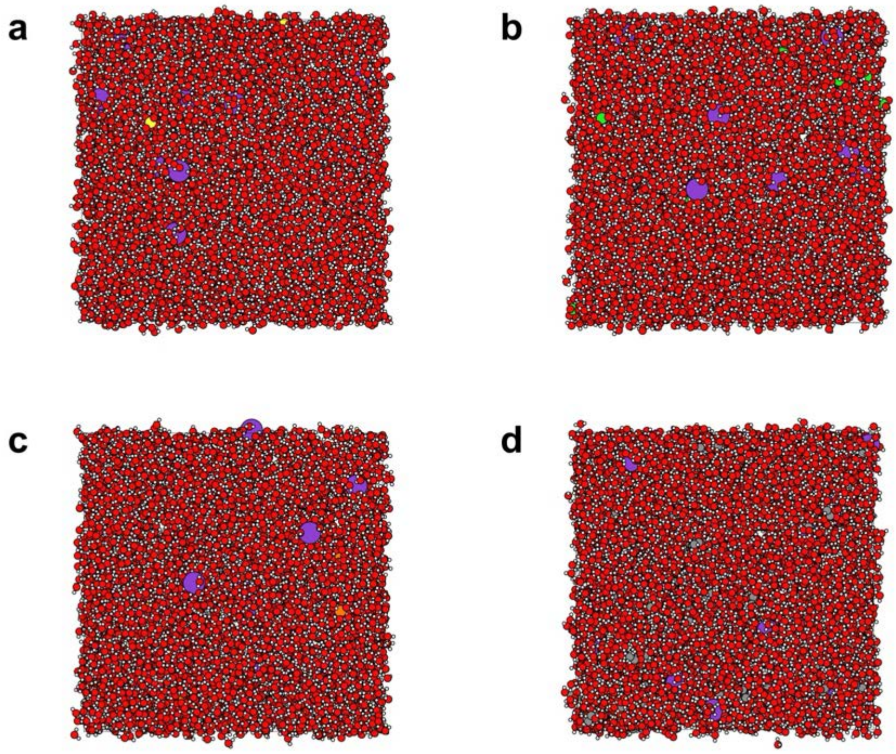

Supplementary Fig. 16 | Snapshots of pH = 4 electrolyte box for MD simulation. (a) 0.05 M K_2SO_4 (H_2SO_4), (b) 0.1 M $KClO_4$ ($HClO_4$), (c) 0.1 M KH_2PO_4 (H_3PO_4), (d) 0.1 M KOAc (HOAc). Red balls stand for oxygen, white balls stand for hydrogen, grey balls stand for carbon, green balls stand for chlorine, yellow balls stand for sulfur, pink balls stand for phosphorus, purple balls stand for potassium. Due to low concentrations (~1E−4 M) at pH = 4, $HClO_4$, H_2SO_4, and H_3PO_4 molecules were excluded from MD simulations in favor of their potassium salts. While HOAc molecules were not excluded from MD simulations due to a higher concentration (~0.57 M).

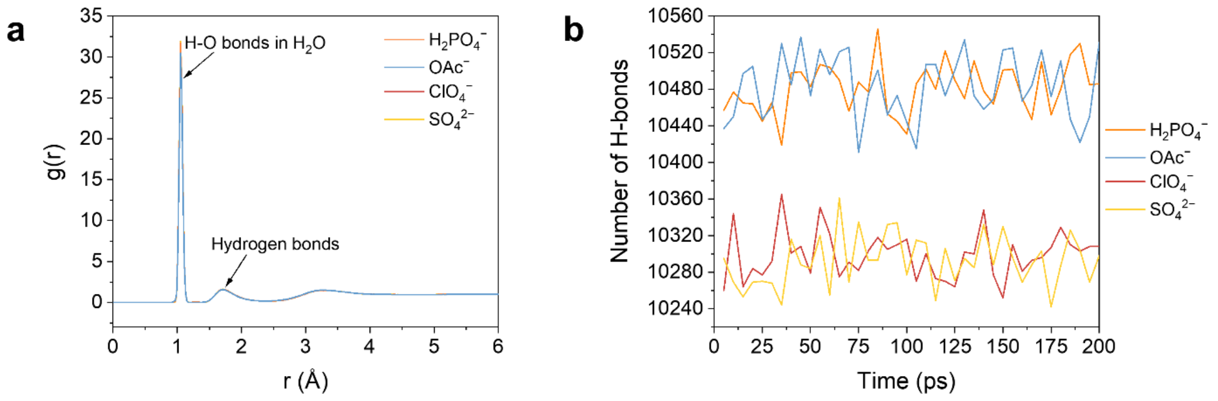

Supplementary Fig. 17 | Calculation of H-bonds number between H_2O in the electrolyte box. (a) Radial distribution functions of H around O in water molecules for different electrolytes, with a cutoff distance of ~2.4 Å to define the maximum hydrogen bond (H-bond) length. (b) Number of H-bonds between water molecules in the electrolyte box over a 200 ps molecular dynamics simulation, with data recorded every 5 ps.

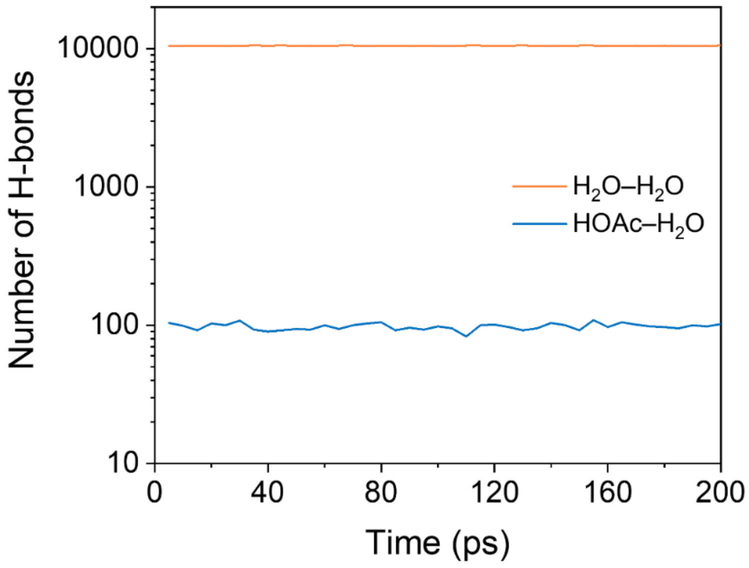

Supplementary Fig. 18 | Comparison of H-bonds between H_2O–H_2O and HOAc–H_2O. Number of H-bonds between H_2O molecules and HOAc molecules in the pH = 4 OAc^--containing electrolyte box over a 200 ps molecular dynamics simulation, with data recorded every 5 ps.

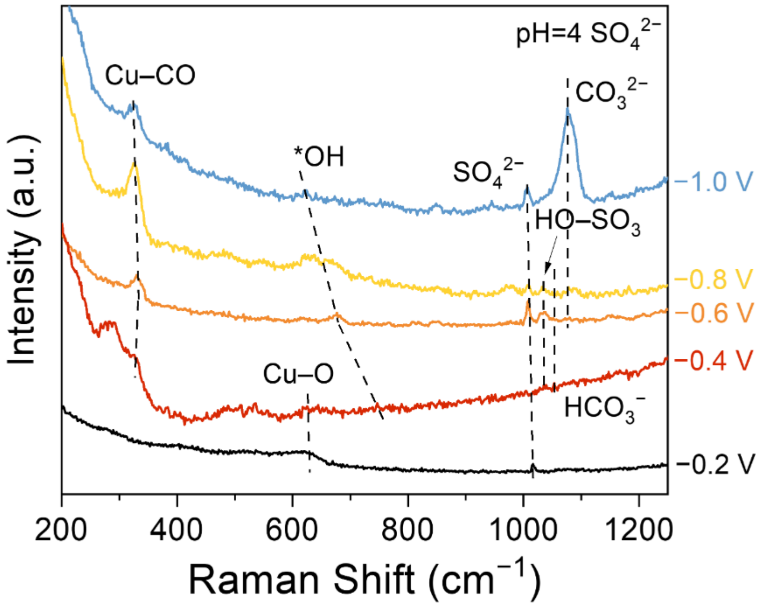

Supplementary Fig. 19 | *In-situ* Raman spectra of ED-Cu in pH = 4 0.05 M K_2SO_4 (H_2SO_4) with key species labeled.

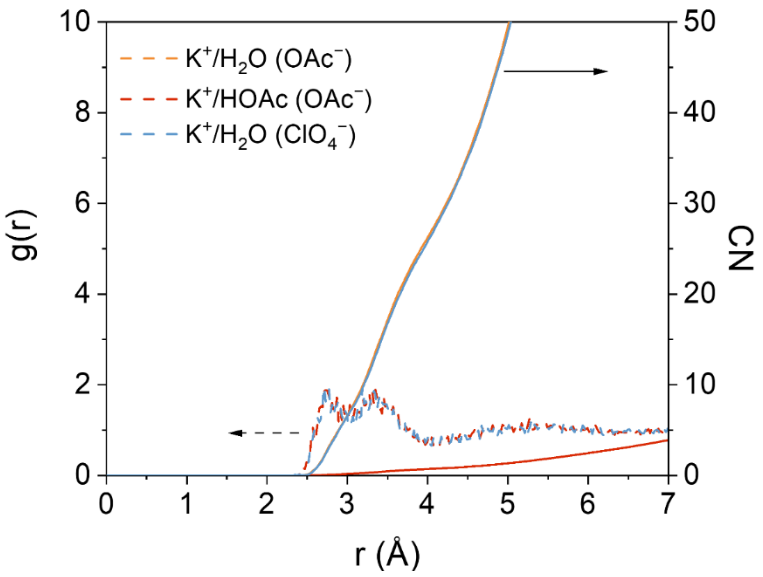

Supplementary Fig. 20 | Calculation of K$^+$ solvation structure in the electrolyte box. Radial distribution functions of HOAc molecules and H_2O molecules around K$^+$ in OAc$^-$-containing electrolyte, along with H_2O molecules around K$^+$ in ClO_4^--containing electrolyte (dashed lines). Corresponding coordination numbers (CN) are shown as solid lines.

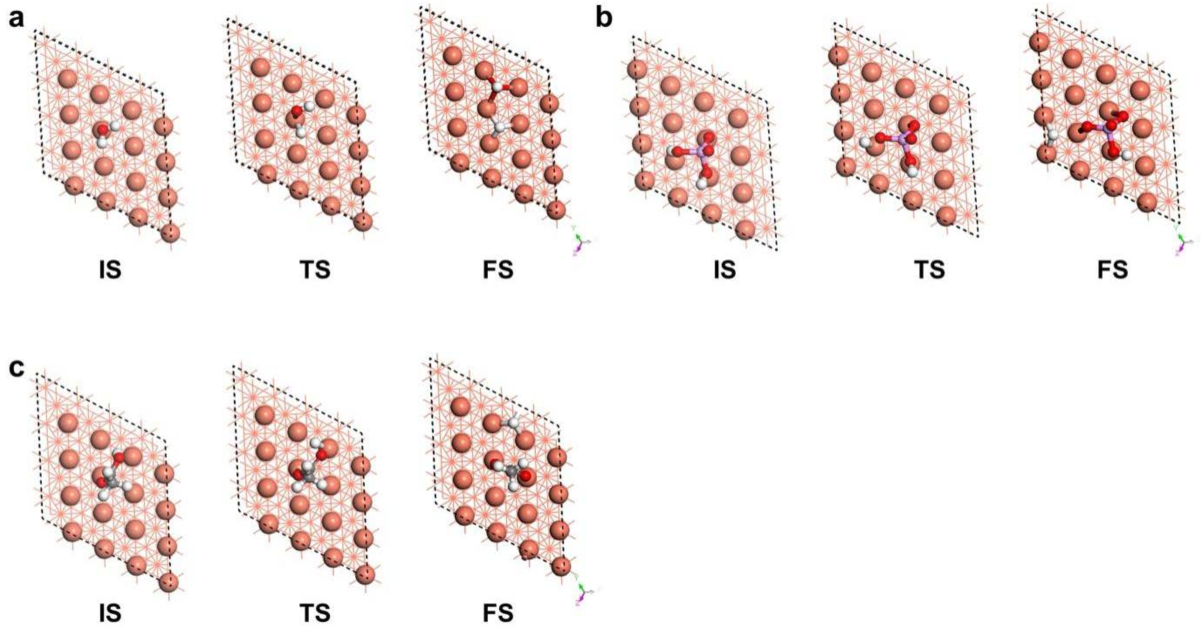

Supplementary Fig. 21 | Atomic structures of the initial state (IS), transition state (TS), and final state (FS) in the kinetic energy diagrams for *H generation from adsorbed (a) H_2O, (b) $H_2PO_4^-$, and (c) HOAc.

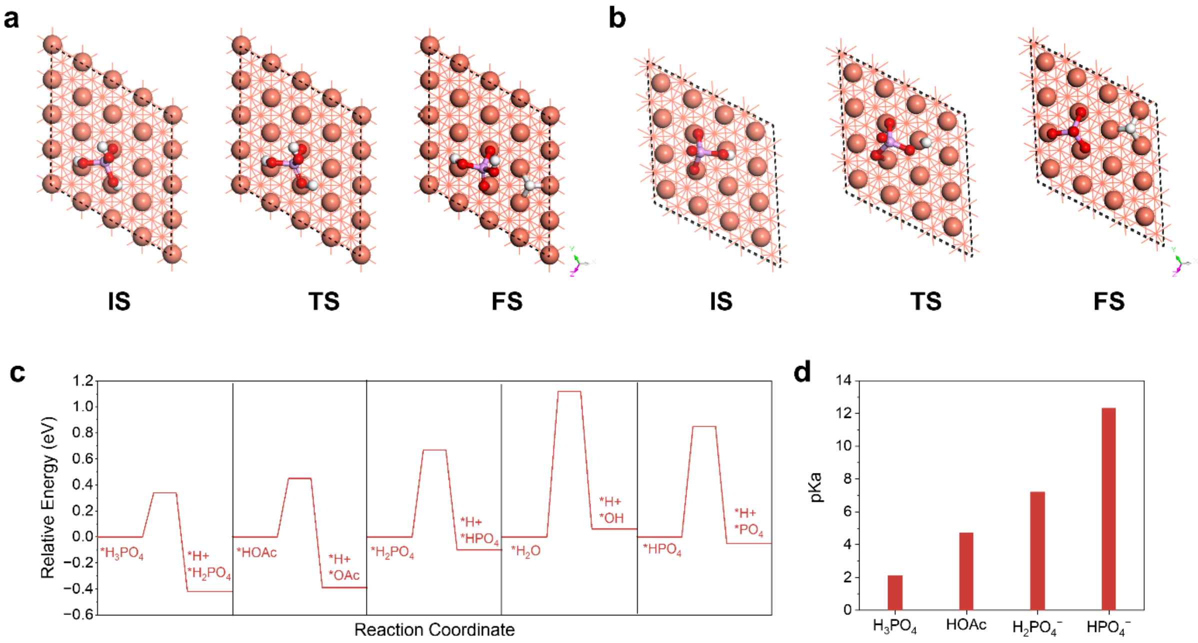

Supplementary Fig. 22 | Kinetics barriers for *H generation at −1.0 V vs RHE with different proton donor. (a–b) Atomic structures of the initial state (IS), transition state (TS), and final state (FS) in the kinetic energy diagrams for H atom transfer from adsorbed (a) H_3PO_4, and (b) HPO_4^{2-}. (c) Kinetic energy diagram showing the dissociation of different protonated species on bare Cu(111) to form the *H intermediate. (d) p*K*a values of different proton donors.

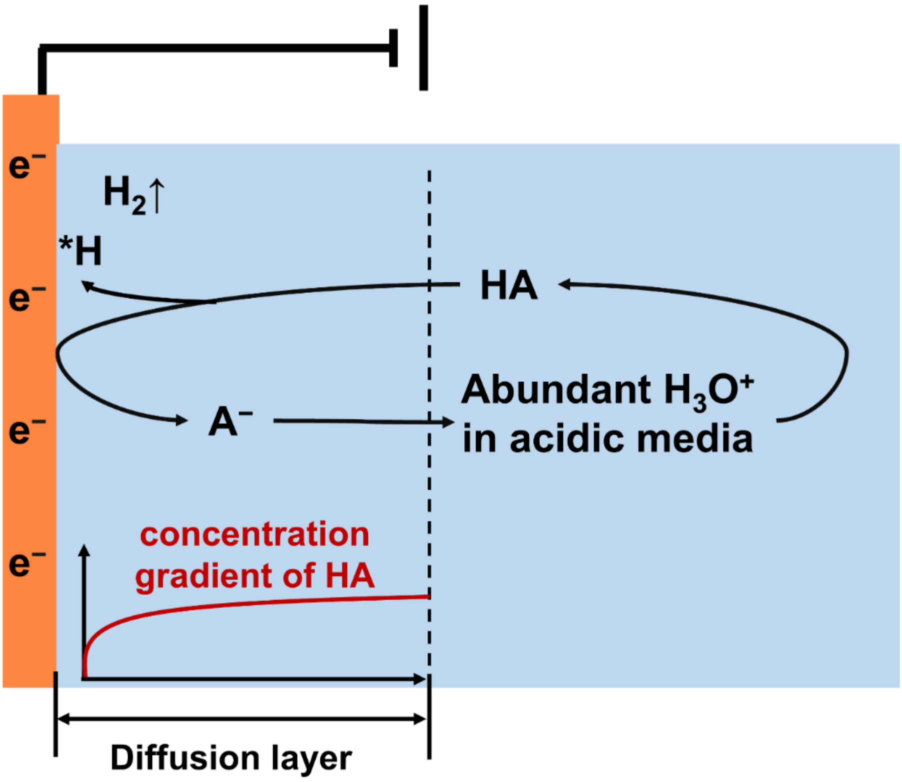

Supplementary Fig. 23 | Schematic of *HA consumption in the diffusion layer at the electrode.

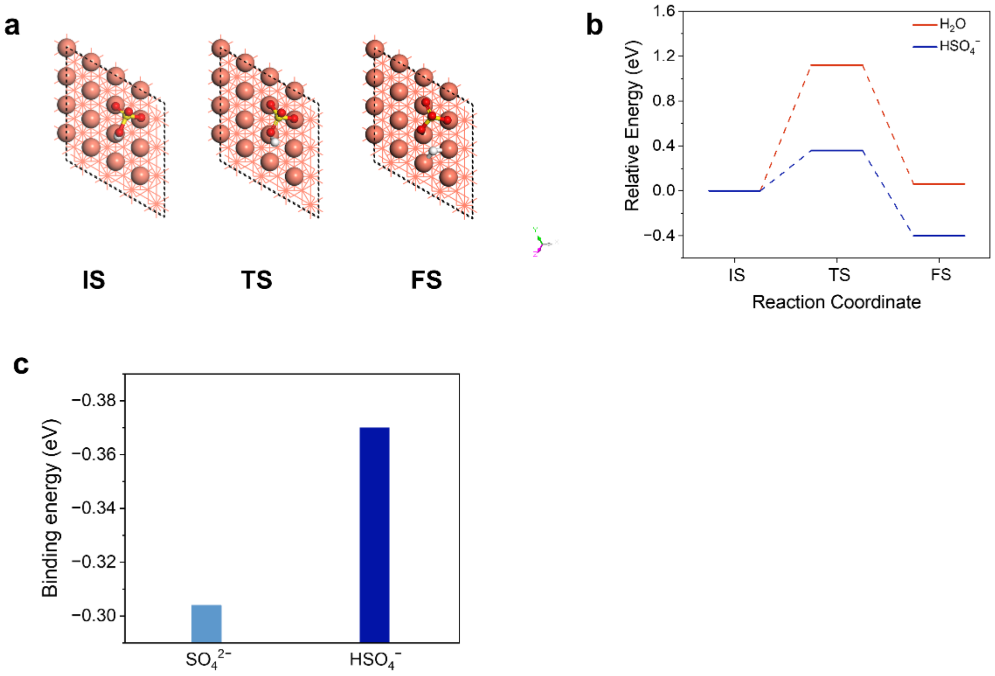

Supplementary Fig. 24 | HSO_4^- improves proton transfer in pH = 2 electrolytes. (a) Atomic structures of the initial state (IS), transition state (TS), and final state (FS) in the kinetic energy diagrams for *H generation from adsorbed H_2O and HSO_4^-. (b) Kinetic energy diagram showing the dissociation on bare Cu(111) to form the *H intermediate at −1.0 V vs RHE. (c) Relative binding energies between water molecules in the presence of SO_4^{2-} and HSO_4^-.

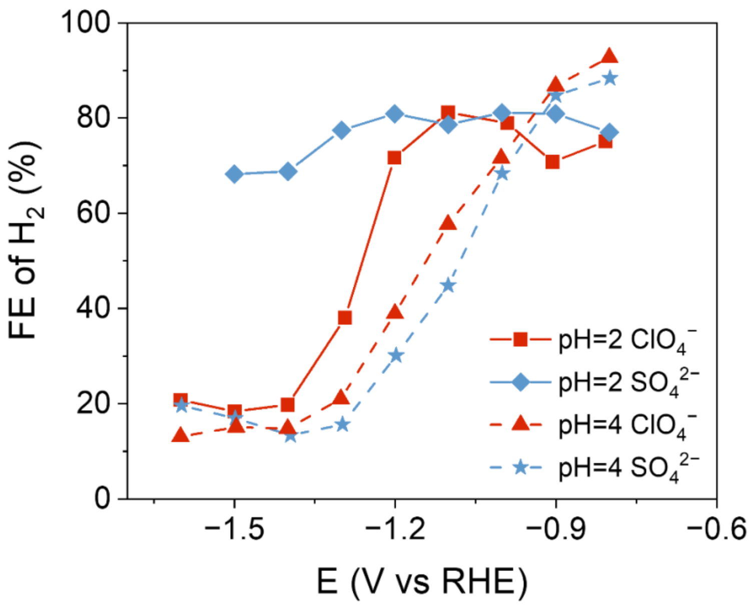

Supplementary Fig. 25 | Calculated FEs of H_2 during LSV in 0.1 M $KClO_4$ ($HClO_4$) and 0.05 M K_2SO_4 (H_2SO_4) at pH = 2 and 4.

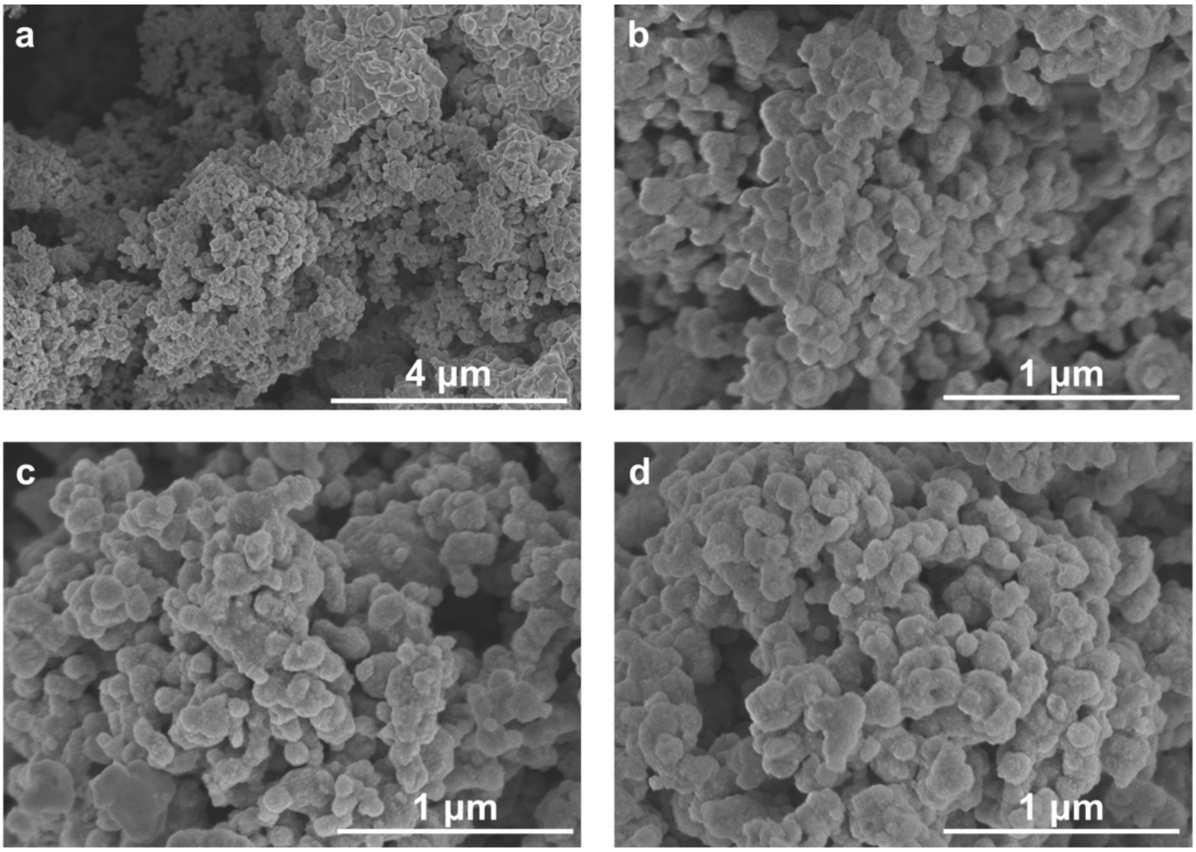

Supplementary Fig. 26 | SEM images of commercial Cu nanoparticles.

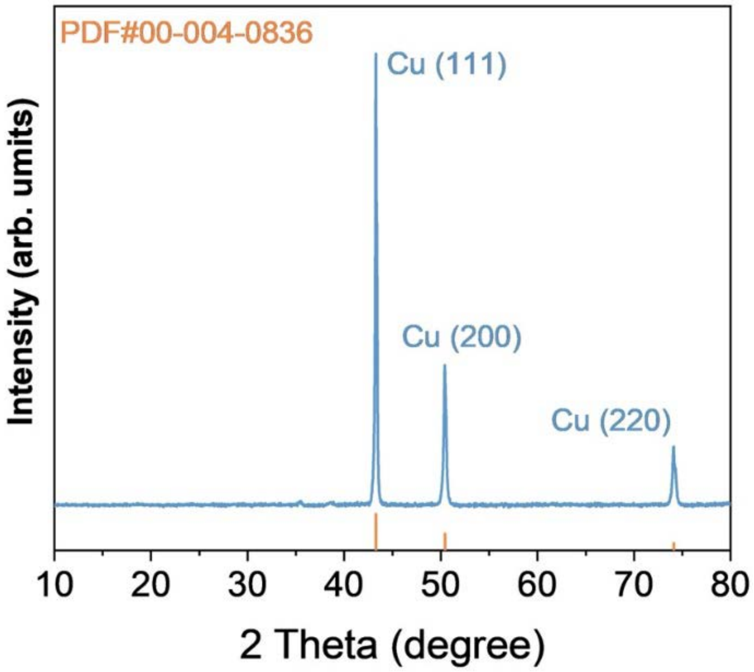

Supplementary Fig. 27 | XRD pattern of commercial Cu nanoparticles.

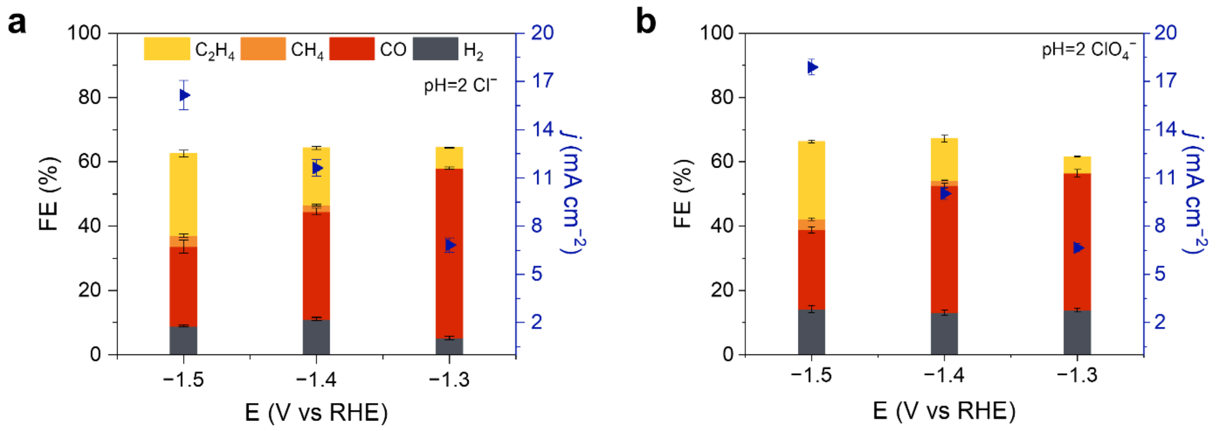

Supplementary Fig. 28 | FEs of gas phase products and total current densities under different applied potentials in (a) pH = 2 0.1 M KCl (HCl), (b) pH = 2, 0.1 M $KClO_4$ ($HClO_4$).

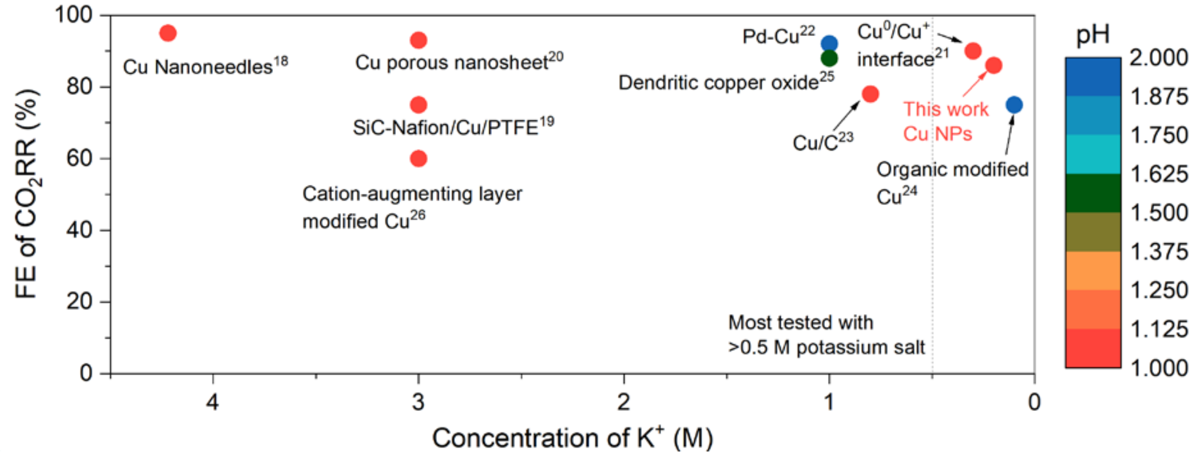

Supplementary Fig. 29 | Comparison of this work with previous studies on the acidic CO_2RR (Refs. [18-26]).

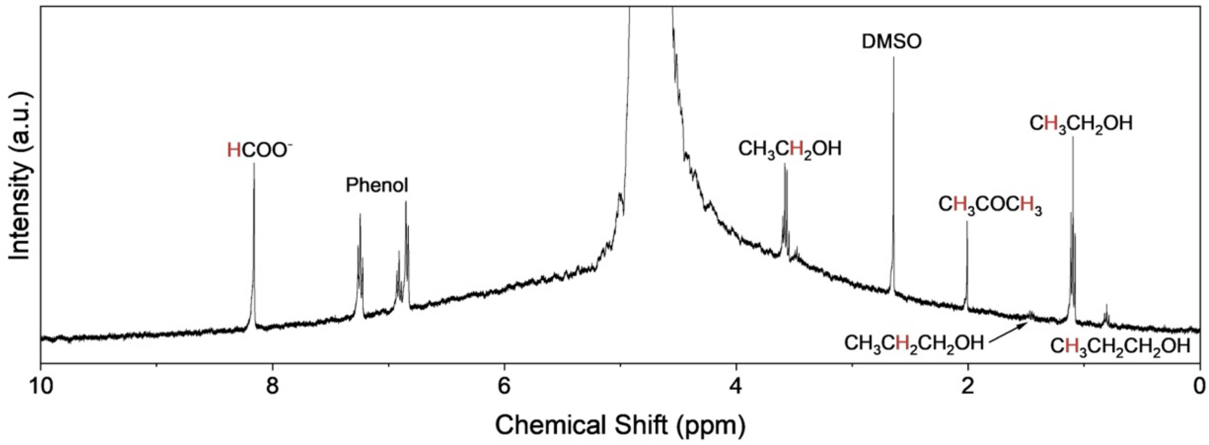

Supplementary Fig. 30 | ^{1}H-NMR spectrum of liquid products of commercial Cu nanoparticles loaded on GDE at 200 mA cm^{-2} after 10 minutes of electrocatalysis in a flow cell.

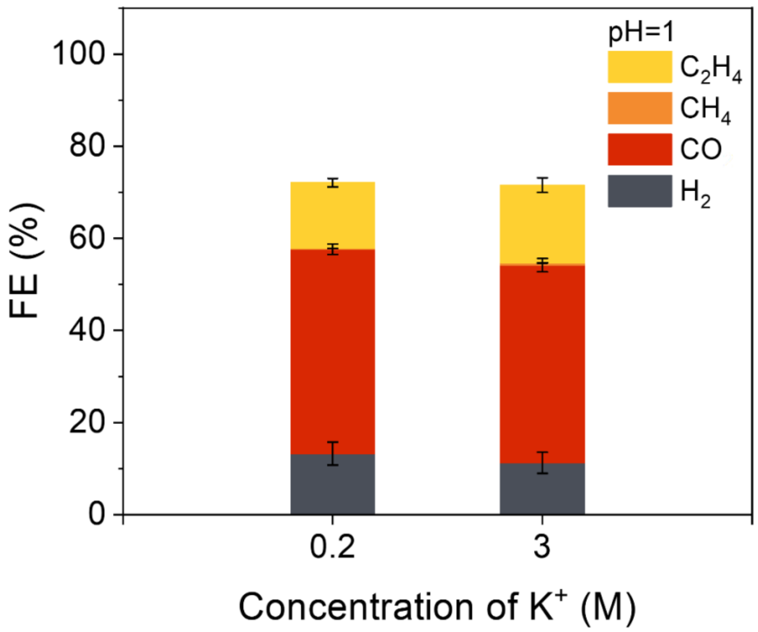

Supplementary Fig. 31 | FEs of gas-phase products in acidic electrolytes (pH = 1) with 0.2 M KCl and 3 M KCl at 100 mA cm^{-2}.

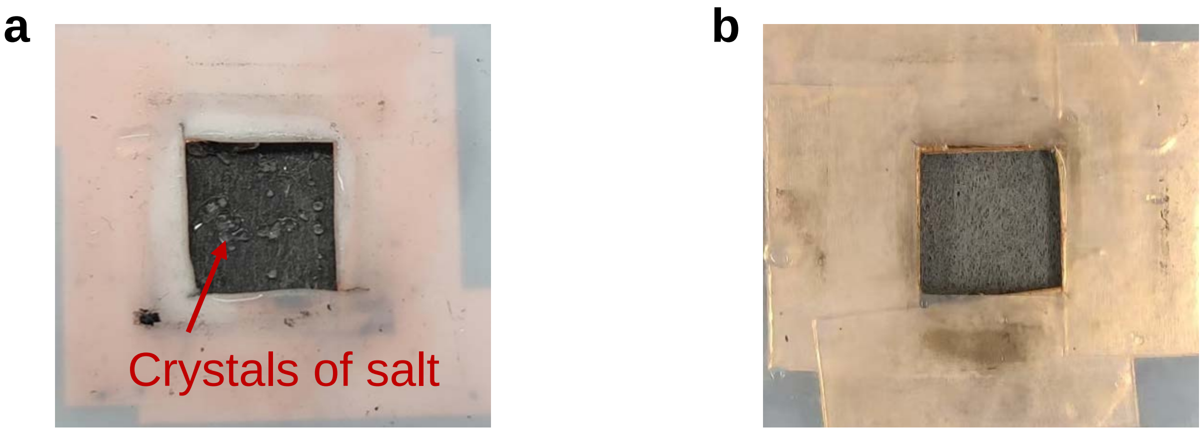

Supplementary Fig. 32 | Digital images of salt precipitation. (a–b) The back side of GDE after electrolysis for 1 hour under 100 mA cm^{-2} in pH = 1 (a) 3 M KCl (HCl), and (b) in 0.2 M KCl (HCl).

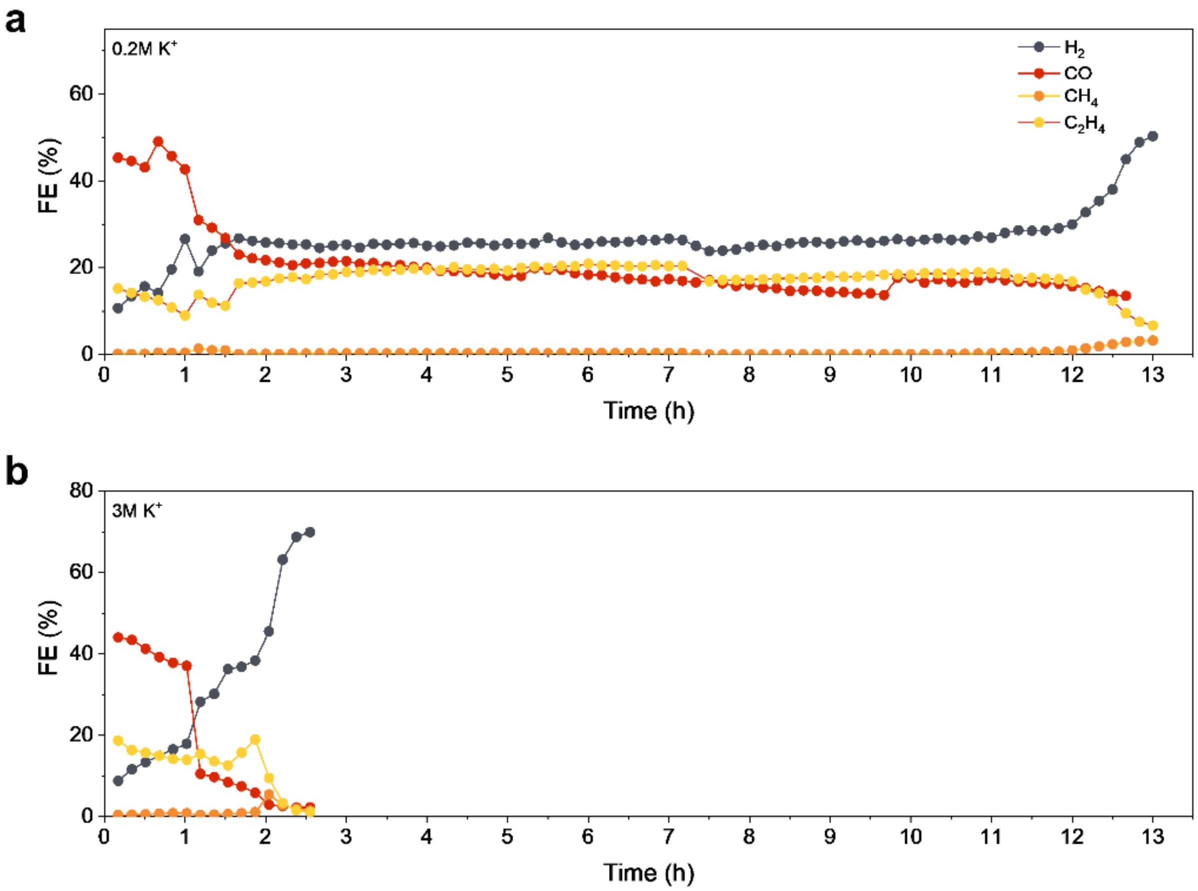

Supplementary Fig. 33 | Stability of electrolyzer with different concentration of KCl. FEs of gas-phase products in acidic (pH = 1) (a) 0.2 M KCl (HCl), (b) 3 M KCl (HCl).

Reference

1. Meunier M. Diffusion coefficients of small gas molecules in amorphous cis-1,4-polybutadiene estimated by molecular dynamics simulations. *J Chem Phys* 2005;**123**:134906.

2. Grimme S, Ehrlich S, Goerigk L. Effect of the damping function in dispersion corrected density functional theory. *J Comput Chem* 2011;**32**:1456–65.

3. Kresse G, Furthmüller J. Efficient iterative schemes for ab initio total-energy calculations using a plane-wave basis set. *Phys Rev B* 1996;**54**:11169–86.

4. Blöchl PE. Projector augmented-wave method. *Phys Rev B* 1994;**50**:17953–79.

5. Grimme S, Antony J, Ehrlich S *et al.* A consistent and accurate ab initio parametrization of density functional dispersion correction (DFT-D) for the 94 elements H-Pu. *J Chem Phys* 2010;**132**, DOI: 10.1063/1.3382344.

6. Mathew K, Sundararaman R, Letchworth-Weaver K *et al.* Implicit solvation model for density-functional study of nanocrystal surfaces and reaction pathways. *J Chem Phys* 2014;**140**, DOI: 10.1063/1.4865107.

7. Mathew K, Kolluru VSC, Mula S *et al.* Implicit self-consistent electrolyte model in plane-wave density-functional theory. *J Chem Phys* 2019;**151**:234101.

8. Zhao X, Liu Y. Origin of Selective Production of Hydrogen Peroxide by Electrochemical Oxygen Reduction. *J Am Chem Soc* 2021;**143**:9423–8.

9. Yu S, Levell Z, Jiang Z *et al.* What Is the Rate-Limiting Step of Oxygen Reduction Reaction on Fe–N–C Catalysts? *J Am Chem Soc* 2023;**145**:25352–6.

10. Henkelman G, Uberuaga BP, Jónsson H. A climbing image nudged elastic band method for finding saddle points and minimum energy paths. *J Chem Phys* 2000;**113**:9901–4.

11. Ye X, Han D, Jiang G *et al.* Unraveling the deposition/dissolution chemistry of MnO_2 for high-energy aqueous batteries. *Energy Environ Sci* 2023;**16**:1016–23.

12. Bondue CJ, Graf M, Goyal A *et al.* Suppression of hydrogen evolution in acidic electrolytes by electrochemical CO_2 reduction. *J Am Chem Soc* 2021;**143**:279–85.

13. Gupta N, Gattrell M, MacDougall B. Calculation for the cathode surface concentrations in the electrochemical reduction of CO_2 in $KHCO_3$ solutions. *J Appl Electrochem* 2006;**36**:161–

72.
14. Resasco J, Lum Y, Clark E *et al.* Effects of anion identity and concentration on electrochemical reduction of CO_2. *ChemElectroChem* 2018;**5**:1064–72.
15. Dinh C-T, Burdyny T, Kibria MG *et al.* CO_2 electroreduction to ethylene via hydroxide-mediated copper catalysis at an abrupt interface. *Science* 2018;**360**:783–7.
16. Singh MR, Clark EL, Bell AT. Effects of electrolyte, catalyst, and membrane composition and operating conditions on the performance of solar-driven electrochemical reduction of carbon dioxide. *Phys Chem Chem Phys* 2015;**17**:18924–36.
17. Handbook of Chemistry and Physics.
18. Zi, X. *et al.* Breaking K^+ concentration limit on Cu nanoneedles for acidic electrocatalytic CO_2 reduction to multi-carbon products. *Angew. Chem. Int. Ed.* **62**, e202309351 (2023).
19. Li L, Liu Z, Yu X *et al.* Achieving High Single-Pass Carbon Conversion Efficiencies in Durable CO_2 Electroreduction in Strong Acids via Electrode Structure Engineering. *Angew Chem Int Ed* 2023;**62**:e202300226.
20. Ma Z, Yang Z, Lai W *et al.* CO_2 electroreduction to multicarbon products in strongly acidic electrolyte via synergistically modulating the local microenvironment. *Nat Commun* 2022;**13**:7596.
21. Jiang Y, Li H, Chen C *et al.* Dynamic Cu_0/Cu^+ Interface Promotes Acidic CO_2 Electroreduction. *ACS Catal* 2024;**14**:8310–6.
22. Xie Y, Ou P, Wang X *et al.* High carbon utilization in CO_2 reduction to multi-carbon products in acidic media. *Nat Catal* 2022;**5**:564–70.
23. Gu J, Liu S, Ni W *et al.* Modulating electric field distribution by alkali cations for CO_2 electroreduction in strongly acidic medium. *Nat Catal* 2022;**5**:268–76.
24. Nie W, Heim GP, Watkins NB *et al.* Organic additive-derived films on Cu electrodes promote electrochemical CO_2 reduction to C_{2+} products under strongly acidic conditions. *Angew Chem Int Ed* 2023;**62**:e202216102.
25. Yang R, Wu M, Huang D *et al.* Which dominates industrial–current–density CO_2-to-C_{2+} electroreduction: $Cu^{\delta+}$ or the microenvironment? *Energy Environ Sci* 2024;**17**:2897–907.
26. Qin H-G, Du Y-F, Bai Y-Y *et al.* Surface-immobilized cross-linked cationic

polyelectrolyte enables CO_2 reduction with metal cation-free acidic electrolyte. *Nat Commun* 2023;**14**:5640.
